# Effects of the Gut Microbiota and Barrier Function on Melatonin Efficacy in Alleviating Liver Injury

**DOI:** 10.3390/antiox11091727

**Published:** 2022-08-31

**Authors:** Hao Zhang, Xiaoyun Liu, Mabrouk Elsabagh, Ying Zhang, Yi Ma, Yaqian Jin, Mengzhi Wang, Hongrong Wang, Honghua Jiang

**Affiliations:** 1 Laboratory of Metabolic Manipulation of Herbivorous Animal Nutrition, College of Animal Science and Technology, Yangzhou University, Yangzhou 225009, China; 2 Joint International Research Laboratory of Agriculture and Agri-Product Safety, The Ministry of Education of China, Yangzhou University, Yangzhou 225009, China; 3 Department of Animal Production and Technology, Faculty of Agricultural Sciences and Technologies, Niğde Ömer Halisdemir University, Nigde 51240, Turkey; 4 Department of Nutrition and Clinical Nutrition, Faculty of Veterinary Medicine, Kafrelsheikh University, Kafrelsheikh 33516, Egypt; 5 Department of Pediatrics, Northern Jiangsu People’s Hospital, Clinical Medical College, Yangzhou University, Yangzhou 225001, China

**Keywords:** cadmium, melatonin, intestinal microbiota, liver injury, gut microbiota transplantation

## Abstract

Environmental cadmium (Cd) exposure has been associated with severe liver injury. In contrast, melatonin (Mel) is a candidate drug therapy for Cd-induced liver injury due to its diverse hepatoprotective activities. However, the precise molecular mechanism by which Mel alleviates the Cd-induced liver injury, as well as the Mel–gut microbiota interaction in liver health, remains unknown. In this study, mice were given oral gavage CdCl_2_ and Mel for 10 weeks before the collection of liver tissues and colonic contents. The role of the gut microbiota in Mel’s efficacy in alleviating the Cd-induced liver injury was evaluated by the gut microbiota depletion technique in the presence of antibiotic treatment and gut microbiota transplantation (GMT). Our results revealed that the oral administration of Mel supplementation mitigated liver inflammation, endoplasmic reticulum (ER) stress and mitophagy, improved the oxidation of fatty acids, and counteracted intestinal microbial dysbiosis in mice suffering from liver injury. It was interesting to find that neither Mel nor Cd administration induced any changes in the liver of antibiotic-treated mice. By adopting the GMT approach where gut microbiota collected from mice in the control (CON), Cd, or Mel + Cd treatment groups was colonized in mice, it was found that gut microbiota was involved in Cd-induced liver injury. Therefore, the gut microbiota is involved in the Mel-mediated mitigation of ER stress, liver inflammation and mitophagy, and the improved oxidation of fatty acids in mice suffering from Cd-induced liver injury.

## 1. Introduction

Cadmium (Cd) has been recognized as a main environmental pollutant due to its extreme toxicity and lack of favorable biological effects on the body [1]. Currently, the Cd level is rapidly increasing in the environment, and consequently, public concerns are arising regarding its possible hazards to human health, particularly in China [2]. The liver is one of the target organs for the bioaccumulation of Cd, which shows high susceptibility to Cd toxicity [3]. Environmental Cd exposure induced the disordered arrangement of hepatic cords and lipid deposition [4]. Hepatic Cd exposure undermines the hepatic immune defense and induces the generation of pro-inflammatory cytokines [5]. In addition, Cd exposure increases diverse stress protein levels and activates hepatocellular damage through the apoptotic or autophagic pathways [6]. Endoplasmic reticulum (ER) stress is reported to be involved in the genesis and progression of Cd-mediated hepatocellular carcinoma [7]. Moreover, the impact of Cd on intestinal microbial dysbiosis [8] and intestinal barrier integrity damage [9] has been elucidated. Gut dysbiosis induces gut inflammation and chronic inflammatory hepatopathy [10]. Anatomically, the gastrointestinal tract is tightly associated with the liver via portal circulation, which is also called the gut−liver axis [10]. Increasing attention has been paid to the influences of xenobiotics on gut microbial structures and functions, as well as on the host physiology [11]. As a result, gut microbiota screening may serve as a valuable tool in the evaluation of environmental pollutant toxicity, especially for trace heavy metals such as Cd. In line with the gut–liver axis theory, gut health plays a key role in hepatopathy occurrence and development [12]. 

Melatonin (Mel) is a circulatory indole hormone generated from the pineal gland and a variety of tissues in mammals, such as the gut, liver and heart [13]. Mel can protect hepatic cells from Cd-induced inflammation and death by suppressing the TXNIP-NLRP3 inflammasome signal pathway [5]. Mel exerts protective effects on mitigating Cd-induced autophagy and hepatotoxicity via the SIRT3-SOD2 signal pathway, in vivo and in vitro [6]. In addition, Mel has a positive effect on the body weight (BW) gain, intestinal ETEC infection and intestinal morphology of weaned mice via modulating the gut microbiota [14]. Therefore, Mel may be a candidate drug therapy for Cd-induced liver injury since it has several hepatoprotective activities. Nonetheless, the exact mechanism of Mel in ameliorating Cd-induced liver injuries, as well as the Mel–gut microbiota interaction in liver health, remains unknown.

It is hypothesized that Mel modulates Cd-induced liver inflammation, ER stress, mitophagy and lipid dysmetabolism in mice via its activities in restoring the intestinal barrier function and rebalancing the gut microbiota profile. This study, therefore, aimed to explore the protective roles of Mel against Cd-induced liver injury and its impacts on gut microbiota and barrier functions.

## 2. Materials and Methods

### 2.1. Animals and Experimental Design

The study protocol was approved by the Committee on the Care and Use of Laboratory Animals of Yangzhou University, China (SXXY 2015-0054). All experimental procedures were conducted according to the Guidelines of Experimental Animal Administration from the State Committee of Science and Technology in China.

The male C57BL/6 (B6, 5 weeks old) mice were provided by the Nanjing Biomedical Research Institute (Nanjing, China). All mice were fed the standard diet of laboratory mice and raised under the following conditions: 12 h/12 h light−dark cycle, 23 ± 2 °C and 50 ± 5% humidity. After one week of adaptation to the diets and rearing conditions, 36 male mice weighing 21.16 ± 0.89 g were randomized into 3 treatment groups, with 12 mice in each group. The 3 treatment groups included (1) control (CON) group: mice received 0.1 M sodium bicarbonate (NaHCO_3_); (2) Cd-treated group: mice received cadmium chloride (CdCl_2_, 10 mg/kg BW) dissolved in 0.1 M NaHCO_3_ [4]; (3) Cd + Mel group: mice received CdCl_2_ (10 mg/kg BW) and Mel (20 mg/kg BW; dissolved in 0.1 M NaHCO_3_ (1 mL)); all mice received treatments once a day for 10 continuous weeks. All treatments were delivered orally using oral gavage of equal volume. CdCl_2_ and Mel were provided by the Sigma–Aldrich Chemical Company (Cat. No. 20,178 and 02518, St. Louis, MO, USA), and their doses were adopted from previous research [15]. During the experiment, mice in all treatment groups were fed ad libitum the laboratory mice standard diet (consisting of 26% wheat flour, 40% corn flour, 10% fish meal, 10% bran, 10% bean cake, 2% minerals, 1% vitamins complex and 1% coarse meal), with free access to clean drinking water. The mean Cd level in the standard diet was approximately 65 ng/g (<1.0% relative to that contained in the CdCl_2_ gavage). At the end of the experiment, each mouse was fasted overnight, followed by euthanization (at 09:00) via cervical dislocation after CO_2_ inhalation [15]. Blood, liver, colon, and ileum samples were collected; blood was sampled from retro-orbital sinus and centrifuged at 3000× *g* under 4 °C for 10 min followed by storage at −20 °C for subsequent assays. The middle segment of the liver (around 1–2 cm) was rinsed with PBS (pH = 7.2–7.4), fixed with 4% paraformaldehyde and embedded within paraffin. One portion of the liver, as well as colonic and ileal mucosal samples, was frozen in liquid nitrogen for extracting mRNA and protein for qRT-PCR and western blotting assays. Colonic contents were also collected and stored at −80 °C until analysis. The body weight (BW) was measured during the experiment.

### 2.2. Oral Gavage of Mel and CdCl_2_ in Antibiotic-Treated Mice

The 6-week-old male mice weighing 21.79 ± 0.76 g were given a standard diet and antibiotic treatment (1 g/L streptomycin, 1 g/L gentamicin, 0.5 g/L ampicillin and 0.5 g/L vancomycin, diluted in drinking water, Meilun Bio, Dalian, China) for 1 week to clean their gut microbiota [14]. Afterwards, the mice were randomized into 3 treatment groups: the antibiotics (anti (CON)), antibiotics + CdCl_2_ (anti (Cd)), and antibiotics + CdCl_2_ + Mel (anti (Cd + Mel)) group (n = 12/group). Total bacterial 16S rRNA was measured through qPCR, which verified the successful depletion of gut microbiota after antibiotic treatment [16]. Identical treatments in CON, Cd and Cd + Mel mice were given to anti (CON), anti (Cd) and anti (Cd + Mel) mice. After 10 weeks of Mel, Cd and antibiotic treatments were given once daily, with each mouse subjected to overnight fasting before it was euthanized. Consequently, samples of liver, blood, colonic content and mucosa and ileal mucosa were collected and stored at −80 ℃ until subsequent analyses.

### 2.3. Gut Microbiota Transplantation (GMT)

A total of 36 gut-microbiota-depleted six-week-old male mice weighing 21.32 ± 0.79 g were used in the GMT trial. The antibiotics-containing water was substituted by the regular water [17]. Mice with depleted gut microbiota and fed with a standard diet of laboratory mice underwent GMT from CON [GMT (CON)], Cd [GMT (Cd) and Mel + Cd [GMT (Mel + Cd)] mice (n = 12 in each group). Then, colonic contents (150 mg) were harvested from CON, Cd and Mel + Cd mice, followed by resuspension within sterile anaerobic saline (2.0 mL), 3 min of vortexing and 2 min of standing by gravity. A total of 0.3 mL of the supernatant obtained from the colonic contents of donor mice was transplanted into the recipient mice through gavage once daily for 10 weeks. At the end of the trial, mice were given CO_2_ inhalation for euthanasia and euthanized through cervical dislocation (at 09:00) to collect blood, liver, colonic content, and mucosa and ileum mucosa samples for subsequent analyses.

### 2.4. Intestinal Morphology Analysis

Ileum fixed with 4% paraformaldehyde was dried through gradient washing with ethanol and xylene, followed by paraffin embedding. Then, 5 slides (5 μm sections were set for each slide) were made from ileal samples. Later, the 5-μm sections were deparaffinized and rehydrated using gradient ethanol and xylene dilutions. To investigate the gut morphology, the slides were stained with hematoxylin and eosin by 20 well-oriented crypts and villi in every section of each slide (Optimus software, version 6.5, Media Cybergenetics). Thereafter, the villi/crypt ratio (VCR) was determined. Five or more crypts and villi in each slide were adopted for quantifying the villus length and crypt depth [18]. 

### 2.5. Biochemical Analysis

The Cd content in the liver and ileum was measured as follows. The wet liver and ileum from each group were precisely collected and dissolved within HNO_3_ to completely remove the bio-organic materials. Then, the Cd contents (μg/g) were measured by inductively coupled plasma mass spectrometry (ICP-MS) (PerkinElmer, Shelton, CT, USA) [4]. Mel, alkaline phosphatase (ALP), albumin (ALB), total cholesterol (TCHO), triglycerides (TG), insulin, glucose, diamine oxidase (DAO), high/low-density lipoprotein (HDL/LDL) and D-lactic acid levels in serum, as well as cytokine (TNF-α, IL-6 and IL-1β) levels in the liver and ileal samples, were determined by the corresponding ELISA kits according to the corresponding instructions (Nanjing Jiancheng Bioengineering Institute, Nanjing, China). The Tissue Carnitine Palmitoyl-transferase 1 (CPT1) Activity Assay Kit was provided by GenMed Scientifics Inc. (Shanghai, China). The Tissue TG Assay Kit was provided by Applygen Technologies Inc. (Beijing, China). The NAD^+^/NADH Quantification Colorimetric Kit (Biovision, Wuhan, China) was used to measure NAD^+^ and NADH to determine the NAD^+^/NADH ratio. The liver ATP contents were determined using the Enhanced ATP Assay Kit (Beyotime). In addition, the commercially available ELISA Kit (Cloud-Clone Crop., Houston, TX, USA) was used to measure LPS content in the liver, colonic content and ileal samples [15]. The liver and ileal tissues were homogenized with 0.15 M NaCl solution (1 mL) followed by 10 min of centrifugation at 1500× *g* under 4 °C. Homogenized liver and ileal tissues were assayed for their maleic dialdehyde (MDA) level, the total antioxidant capacity (T-AOC) content, and superoxide dismutase (SOD) and glutathione peroxidase (GSH-Px) activities using commercial ELISA kits (Jiancheng Bioengineering Institute, Nanjing, Jiangsu, China) [19].

### 2.6. Ileal Barrier Function Measurements by Ussing Chamber

The ileal barrier function was measured using Ussing chamber analysis based on a previous protocol [17]. Specifically, the intestinal segments were stripped from the seromuscular layer and were loaded into the EasyMount chamber system (model VCC MC6, Physiologic Instruments, San Diego, CA, USA). Notably, there were clamps attached to the Acquire and Analyse software (Physiologic Instruments, San Diego, CA, USA) to automatically collect data. At the same time, transepithelial electrical resistance (TER) was obtained every 15 min within 1 h after Ussing chamber equilibration for 15 min. The paracellular flux of dextran (FD4) level was measured using a microplate reader (FLx800; BioTek Instruments, Inc., Winooski, VT, USA).

### 2.7. Hepatocyte Isolation and Determination of Necrosis and Apoptosis

Hepatocytes were separated from a portion of fresh liver samples to determine the apoptosis and necrosis rates. As detailed, all hematopoietic cells were eliminated while separating the hepatocytes. There were more than 90% classical hepatocytes, and the remaining 10% were fibroblast-like cells. The harvested liver was immediately immersed in the pre-chilled Hanks’ balanced salt solution (HBSS). Then, about 3 g of liver samples were weighed, minced and shaken in the 25 mL HBSS consisting of 5 mM-EDTA for 5 min under 37 ℃. Then, samples were subjected to an additional 10 min of shaking in 25 mL HBSS that contained 5 mM-CaCl_2_, 0.25% (*w/v*) collagenase I and 0.01% (*w/v*) DNase (Sigma-Aldrich) under 37 ℃. After discarding the supernatants, the above process was repeated. Later, the cell suspension was filtered using the 70 mm nylon mesh and centrifuged at 20× *g* for 1 min. Each pellet was further resuspended within PBS and centrifuged for 1 min at 20× *g*. The cell viability was measured through Trypan blue staining [20]. The apoptotic and necrotic rates were measured by differential staining using Annexin V (which stained the apoptotic and necrotic cells) and propidium iodide (PI, stained necrotic cells alone) by adopting the Alexa Fluor^®^ 488-Annexin V/Dead Cell Apoptosis Kit (V13241; Invitrogen Life Technologies, Shanghai, China) according to a previous description [21].

### 2.8. Liver Mitochondria Isolation, ROS and Mitochondria Membrane Potential (ΔΨm) Determination, and the Activities of Respiratory Chain Complexes I–IV

Freshly prepared liver tissues were adopted to prepare mitochondria. Each procedure was conducted following the specific mitochondrial isolation kit instructions (Beyotime Institute of Biotechnology, Nanjing, China). The ROS generated by liver mitochondria were determined through the 20 min incubation of mitochondrial pellets using 2 μmol/L 2′,7′-dichlorohydro-fluorescein diacetate under 24 °C, and then the fluorescence intensity was determined using a fluorescence microplate reader as previously described [22].

The ΔΨm assay kits were used for determining ΔΨm according to the manufacturer’s instructions (Beyotime Institute of Biotechnology, China). In the case of high mitochondrial membrane potential (MMP), JC-1 will aggregate at the mitochondrial matrix to produce a J-aggregate polymer, and it generates red fluorescence. For a low MMP, JC-1 is unable to aggregate into mitochondria. JC-1 serves as a monomer within the matrix at a specific moment which generates green fluorescence. The fluorescence ratio of aggregate to monomer was determined to measure the hepatic mitochondrial ΔΨm by a fluorescence microplate reader (Bio-Tek Instruments, Inc., VT, USA) [23].

The NADH ubiquinone reductase, succinate ubiquinone reductase, ubiquinol cytochrome c reductase and cytochrome c oxidase (namely, complexes I–IV, respectively) activities were assayed based on the method of Hargreaves et al. [24].

### 2.9. Short-Chain Fatty Acids (SCFA)

The butyric, isobutyric, acetic, isovaleric, valeric and propionic acid concentrations in the colonic contents were determined using Agilent 6890 gas chromatography (Agilent Technologies, Santa Clara, CA, USA) according to a previous description [25].

### 2.10. Gut Microbiota Analysis

Specific primers that had respective barcodes (16S V3 + V4) were used to extract the total genomic DNA from the colonic content to carry out amplification. The Illumina MiSeq platform was applied in paired-end sequencing, whereas the Mothur Bayesian classifier was employed to obtain the OUT table and the phylogenic tree. The sequencing libraries were produced and examined following previous research [26]. The principal coordinates were acquired by principal coordinate analysis (PCoA) and were visualized based on complex and multidimensional data. Thereafter, for the observed species, the Simpson, Shannon, ACE and Chao1 indexes were adopted for evaluating the complicated species diversity [17]. OTUs were also used in predicting the microbial community genome using PICRUSt (Phylogenetic Investigation of Communities by Reconstruction of Unobserved States) [27].

### 2.11. Gene Expression Analysis

Total RNA was extracted from the liquid-nitrogen-frozen liver and ileal samples using a TRIZOL reagent (Invitrogen, Carlsbad, CA, USA), followed by DNase I (Invitrogen) treatment following specific protocols. The cDNA was synthesized using the Superscript II reverse transcriptase and oligo (dT) 20 (Invitrogen). Teal-time PCR was performed using the ABI 7900 PCR system (ABI Biotechnology, Eldersburg, MD, USA). The primers were screened based on prior reports (Table S1), with β-actin being the endogenous reference for normalizing the relative expression of target genes. The relative expression of target genes was determined through the 2^−ΔΔCt^ method [17].

### 2.12. Western Blot Analysis

The liver and ileal samples were ground and lysed using a high-efficiency RIPA lysis buffer (Beijing Solarbio Science & Technology Co Ltd., Beijing, China) followed by high-speed centrifugation to extract total tissue proteins. The BCA protein assay kit (Beijing Solarbio Science & Technology Co., Ltd., Beijing, China) was used to detect protein content and made the sample solution (50 μg/20 μL) for subsequent tests. To separate the protein, 8–15% SDS-PAGE was used (10–50 μg per well), which was then transferred to a PVDF membrane. Later, the membranes were blocked using the 1% BSA blocking buffer (Beijing Solarbio Science & Technology Co., Ltd., Beijing, China) for 2 h, followed by 2 h of incubation using the respective primary antibodies and another 1 h incubation using HR-conjugated secondary antibodies (Table S2). Besides, the ECL luminescence reagent was used in the measurement. The expression of target proteins was quantitatively analyzed using the image analysis system (Super ECL Plus, Applygen, Beijing, China). β-actin or VDAC were the reference proteins used to normalize the result of target proteins. At last, the digital quantification of protein signals and normalization was conducted based on β-actin or VDAC [25]. Each densitometry value was normalized according to VDAC or β-actin and expressed as the level relative to the CON value.

### 2.13. Statistical Analysis

The statistical analysis of data was performed using a one-way ANOVA of SPSS19.0 (SPSS Inc., Chicago, IL, USA) followed by Duncan’s multiple range test to detect significant differences among treatments. The values were expressed as means ± SEM, and a *p*-value < 0.05 was deemed to be statistically significant.

## 3. Results

### 3.1. Growth Performance, Serum Enzyme Activities and Lipid Metabolic Indices, Antioxidative Capacity, and LPS and Cytokine Contents

Body weight gain, serum ALB, liver CPT1, and GSH-Px and SOD activities increased (*p* < 0.05), whereas liver weight, liver index, serum ALP, TG, TCHO, LDL, glucose, insulin, HOMA-IR, liver TG, MDA, LPS and cytokine concentrations (IL-6 and TNF-α) decreased (*p* < 0.05) in the Mel + Cd and GMT (Mel + Cd) groups compared to those in the Cd- and GMT (Cd)-treated mice, respectively (Tables S3 and S4). The activities of serum enzymes, growth performance of mice, lipid metabolic indices, liver cytokine contents, LPS level and antioxidative capacity did not change (*p* > 0.05) in antibiotic-treated mice subjected to oral gavage of CdCl_2_ and Mel (Table S5).

### 3.2. Mitochondrial ROS Generation, ΔΨm, Mitochondrial Complex Activities, Apoptosis Levels, NAD^+^/NADH Ratio and ATP Levels

The ROS levels and necrotic and apoptotic cell proportions decreased (*p* *<* 0.05), whereas NAD^+^/NADH, ΔΨm, ATP levels, and complex I/IV activities increased (*p* *<* 0.05) in the Mel + Cd and GMT (Mel + Cd) groups compared to those in the Cd and GMT (Cd) groups, respectively (Tables S6 and S7). On the other hand, the oral gavage of CdCl_2_ and Mel did not induce (*p* > 0.05) any alterations in liver mitochondrial ROS generation, ΔΨm, NAD^+^/NADH, mitochondrial complex activities, ATP levels and apoptotic cell proportion in antibiotic-treated mice (Table S8).

### 3.3. Mel Level in the Serum and Cd Residues in the Liver and Ileum

Compared to Cd- and GMT (Cd)-treated mice, the Mel + Cd and GMT (Mel + Cd) treatments increased serum Mel contents (*p* *<* 0.05) but decreased Cd residues in liver and ileum, respectively (*p* *<* 0.05) (Tables S9 and S10). Compared to mice in the anti (Cd) group, the oral gavage of Mel elevated the serum Mel content (*p* *<* 0.05) but did not alter (*p* > 0.05) Cd residues in the liver and ileum (Table S11).

### 3.4. Villus Morphology, Barrier Function, Anti-Oxidative Capacity, LPS and Cytokine Contents in the Ileum and D-Lactic Acid Content, and DAO Activity in Serum

The TER, VCR, villus height and the activity of SOD, T-AOC and GSH-Px increased (*p* *<* 0.05), whereas crypt depth, FD4 flux, MDA content, LPS and cytokine concentrations, (TNF-α and IL-6) as well as the DAO and D-lactic acid contents, decreased (*p* *<* 0.05) in the ileum and serum of the Mel + Cd- and GMT (Mel + Cd)-treated mice compared to those in Cd- and GMT (Cd)-treated groups, respectively (Tables S12 and S13). The oral gavage of CdCl_2_ and Mel did not modify (*p* > 0.05) villus morphology, barrier function, anti-oxidative capacity, or cytokine and LPS contents in the ileum, or the DAO and D-lactic acid contents in the serum of antibiotic-treated mice (Table S14).

### 3.5. LPS and SCFAs Levels in the Colonic Contents

The LPS levels were reduced (*p* < 0.05), while those of valerate and propionate were elevated in colonic content (*p* < 0.05) from the Mel + Cd and GMT (Mel + Cd) groups compared to those from the Cd and GMT (Cd) groups (Tables S15 and S16). Further, the LPS and SCFA levels in the colonic content from antibiotic-treated mice were not altered (*p* > 0.05) by the oral gavage of CdCl_2_ and Mel (Table S17).

### 3.6. mRNA Levels in the Hepatic and Ileal Tissues

**Figure 1:**
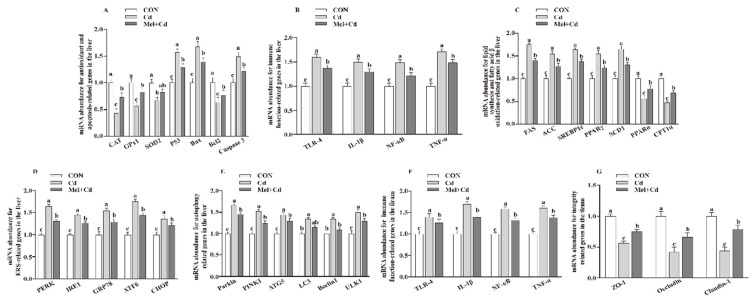
Impact of Mel supplementation on mRNA abundance of genes in the liver and ileum of mice treated with CdCl_2_ for 10 weeks. The mRNA abundance of relative genes in apoptosis and antioxidation (**A**), immune function (**B**), fatty acid oxidation and lipid metabolism (**C**), ERS (**D**), autophagy (**E**) in the liver, and immune function (**F**) and integrity (**G**) in the ileum. ATG5, autophagy related 5; ACC, Acetyl-CoA carboxylase; ATF6, activating transcription factor 6; Bcl-2, B-cell lymphoma/leukaemia 2; Bax, Bcl-2-associated X protein; CON, mice fed with the control diet; CdCl_2_, cadmium chloride; Cd group, mice given gavage of CdCl_2_ once daily; CAT, catalase; CHOP, CCAAT/enhancer-binding protein homologous protein; CPT1α, carnitine palmitoyltransferase 1α; ERS, endoplasmic reticulum stress; FAS, fatty acid synthase; GPx1, glutathione peroxidase 1; GRP78, glucose-regulated protein 78; IL, interleukin; IRE1, inositol-requiring enzyme 1; LC3, microtubule-associated protein light chain 3; Mel + Cd group, mice were treated with Mel and CdCl_2_; Mel, melatonin; NF-κB, nuclear factor kappa B (p65); PERK, protein kinase R-like endoplasmic reticulum kinase; PPAR, peroxisome proliferator-activated receptor; PINK1, PTEN induced putative kinase 1; SOD2, superoxide dismutase 2; SCD-1, stearoyl-CoA desaturase-1; SREBP-1c, Sterol regulatory element binding protein-1c; TNF-α, tumor necrosis factor α; TLR, toll-like receptor; ULK1, unc-51-like autophagy activating kinase 1; ZO-1, zonula occludens-1. Data are expressed as means ± standard errors and presented in the vertical bars (n = 12). Labeled means without a common letter differ (*p <* 0.05).

**Figure 2:**
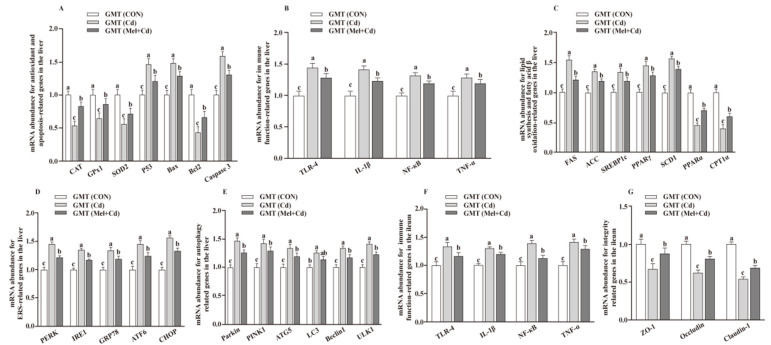
Impacts of GMT from CON, Cd and Mel + Cd mice on mRNA abundance of genes in liver and ileum in antibiotics-treated mice for 10 weeks. The mRNA abundance of relative genes in apoptosis and antioxidation (**A**), immune function (**B**), fatty acid oxidation and lipid metabolism (**C**), ERS (**D**), autophagy (**E**) in the liver, and immune function (**F**) and integrity (**G**) in the ileum. ATG5, autophagy related 5; ACC, Acetyl-CoA carboxylase; ATF6, activating transcription factor 6; Bcl-2, B-cell lymphoma/leukaemia 2; Bax, Bcl-2-associated X protein; CAT, catalase; CHOP, CCAAT/enhancer-binding protein homologous protein; CPT1α, carnitine palmitoyltransferase 1α; ERS, endoplasmic reticulum stress; FAS, fatty acid synthase; CdCl_2_, cadmium chloride; ERS, endoplasmic reticulum stress; GMT, gut microbiota transplantation; GMT(CON), the microbiota-depleted mice received microbiota transplantations from donor mice fed control diet; GMT(Cd), mice with gut microbiota depletion received GMT from CdCl_2_-treated mice; GMT (Mel + Cd), mice with microbial depletion received GMT from Mel + Cd mice; GPx1, glutathione peroxidase 1; GRP78, glucose-regulated protein 78; IL, interleukin; IRE1, inositol-requiring enzyme 1; LC3, microtubule-associated protein light chain 3; Mel, melatonin; NF-κB, nuclear factor kappa B (p65); PERK, protein kinase R-like endoplasmic reticulum kinase; PPAR, peroxisome proliferator-activated receptor; PINK1, PTEN induced putative kinase 1; SOD2, superoxide dismutase 2; SCD-1, stearoyl-CoA desaturase-1; SREBP-1c, Sterol regulatory element binding protein-1c; TNF-α, tumor necrosis factor α; TLR, toll-like receptor; ULK1, unc-51-like autophagy activating kinase 1; ZO-1, zonula occludens-1. Data are expressed as means ± standard errors and presented in the vertical bars (n = 12). Labeled means without a common letter differ (*p <* 0.05).

**Figure 3:**
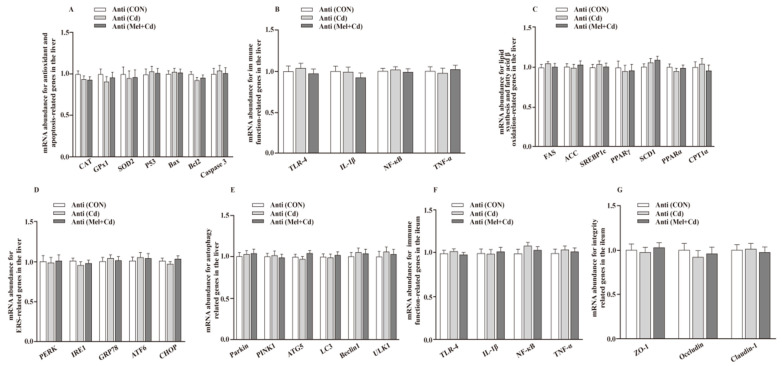
Impacts of Mel and CdCl_2_ oral gavage on mRNA abundance of genes in liver and ileum in antibiotics-treated mice for 10 weeks. The mRNA abundance of relative genes in apoptosis and antioxidation (**A**), immune function (**B**), fatty acid oxidation and lipid metabolism (**C**), ERS (**D**), autophagy (**E**) in the liver, and immune function (**F**) and integrity (**G**) in the ileum. Anti, antibiotics; Anti (CON), mice with gut microbiota depletion; Anti (Cd), mice with gut microbiota depletion were treated with CdCl_2_; Anti (Mel + Cd), mice with gut microbiota depletion were treated with CdCl_2_ and Mel; ATG5, autophagy related 5; ACC, Acetyl-CoA carboxylase; ATF6, activating transcription factor 6; Bcl-2, B-cell lymphoma/leukaemia 2; Bax, Bcl-2-associated X protein; CdCl_2_, cadmium chloride; CAT, catalase; CHOP, CCAAT/enhancer-binding protein homologous protein; CPT1α, carnitine palmitoyltransferase 1α; ERS, endoplasmic reticulum stress; FAS, fatty acid synthase; GPx1, glutathione peroxidase 1; GRP78, glucose-regulated protein 78; IL, interleukin; IRE1, inositol-requiring enzyme 1; LC3, microtubule-associated protein light chain 3; Mel, melatonin; NF-κB, nuclear factor kappa B (p65); PERK, protein kinase R-like endoplasmic reticulum kinase; PPAR, peroxisome proliferator-activated receptor; PINK1, PTEN induced putative kinase 1; SOD2, superoxide dismutase 2; SCD-1, stearoyl-CoA desaturase-1; SREBP-1c, Sterol regulatory element binding protein-1c; TNF-α, tumor necrosis factor α; TLR, toll-like receptor; ULK1, unc-51-like autophagy activating kinase 1; ZO-1, zonula occludens-1. Data are expressed as means ± standard errors and presented in the vertical bars (n = 12).

The hepatic mRNA expression of P53, ACC, Bax, FAS, PPARγ, Caspase 3, SREBP-1c, SCD1, ULK1, ATG5, Beclin1, PINK1, Parkin, PERK, IRE1, GRP78, ATF6 and CHOP, and the hepatic and ileal mRNA expression of TLR-4, IL-1β, NF-κB and TNF-α, decreased (*p* *<* 0.05), whereas the hepatic mRNA expression of GPx1, CAT, CPT1α and PPARα and the ileal mRNA expression of ZO-1, Claudin-1 and Occludin increased (*p* *<* 0.05) in the Mel + Cd and GMT (Mel + Cd) groups compared to those in the Cd and GMT (Cd) groups (Figure 1 and Figure 2). On the other hand, the mRNA levels of apoptotic, antioxidant, immune, ER stress, autophagy, lipid-metabolism- and fatty-acid-oxidation-associated genes in the liver, or those of integrity and immune-associated genes in the ileum, were not changed (*p* > 0.05) by the oral gavage of CdCl_2_ and Mel in mice treated with antibiotics (Figure 3).

### 3.7. Relative Protein Levels in the Hepatic and Ileal Tissues

**Figure 4:**
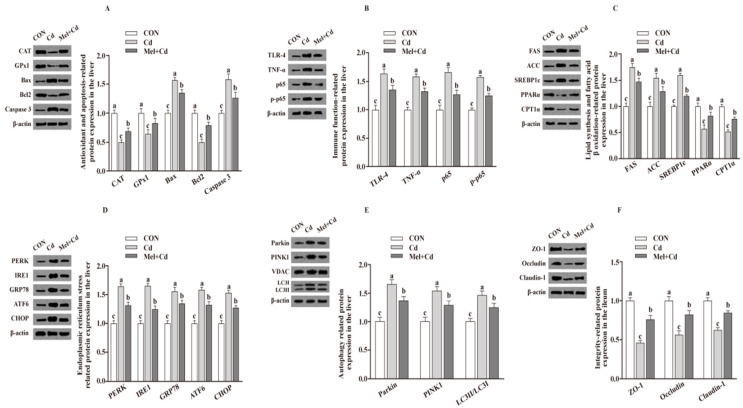
Impact of Mel supplementation on the abundance of proteins in liver and ileum in CdCl_2_-treated mice for 10 weeks. The expression levels of relative proteins in antioxidation and apoptosis (**A**), immune function (**B**), fatty acid oxidation and lipid metabolism (**C**), endoplasmic reticulum stress (**D**), autophagy (**E**) in the liver, and integrity (**F**) in the ileum. ACC, Acetyl-CoA carboxylase; ATF6, activating transcription factor 6; Bcl-2, B-cell lymphoma/leukaemia 2; Bax, Bcl-2-associated X protein; CdCl_2_, cadmium chloride; CON group, mice fed with the control diet; Cd group, mice given gavage of CdCl_2_ once daily; CPT1α, carnitine palmitoyl transferase 1α; CAT, catalase; CHOP, CCAAT/enhancer-binding protein homologous protein; FAS, fatty acid synthase; GRP78, glucose-regulated protein 78; GPx1, glutathione peroxidase 1; IRE1, inositol-requiring enzyme 1; LC3, microtubule-associated protein light chain 3; Mel + Cd group, mice given Mel and CdCl_2_ treatment; Mel, melatonin; NF-κB, nuclear factor kappa B (p65); PINK1, PTEN-induced putative kinase 1; PERK, protein kinase R-like endoplasmic reticulum kinase; PPARα, peroxisome proliferator-activated receptor α; SOD2, superoxide dismutase2; SREBP-1c, Sterol regulatory element-binding protein-1c; TLR, toll-like receptor; TNF-α, tumor necrosis factor α; VDAC, voltage-dependent anion channel; ZO-1, zonula occludens-1. The western blot image is on the left of each bar chart panel. The protein expression value = densitometry units of selected protein/densitometry units of β-actin or VDAC detected by western blotting. β-actin or VDAC was the reference protein to normalize the result of target proteins. Data are expressed as means ± standard errors and presented in the vertical bars (n = 12). Labeled means without a common letter differ (*p* < 0.05).

**Figure 5:**
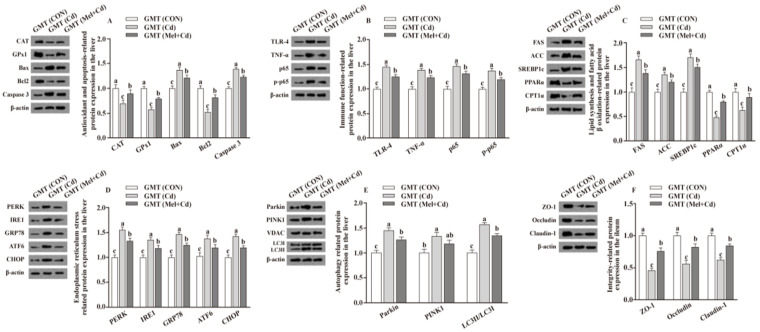
Impact of GMT from CON, Cd and Mel + Cd mice on the abundance of proteins in liver and ileum in antibiotics-treated mice for 10 weeks. The expression levels of relative proteins in antioxidation and apoptosis (**A**), immune function (**B**), fatty acid oxidation and lipid metabolism (**C**), endoplasmic reticulum stress (**D**), autophagy (**E**) in the liver, and integrity (**F**) in the ileum. ACC, Acetyl-CoA carboxylase; ATF6, activating transcription factor 6; Bcl-2, B-cell lymphoma/leukaemia 2; Bax, Bcl-2-associated X protein; CPT1α, carnitine palmitoyl transferase 1α; CAT, catalase; CHOP, CCAAT/enhancer-binding protein homologous protein; CdCl_2_, cadmium chloride; FAS, fatty acid synthase; GMT, gut microbiota transplantation; GMT(CON), the microbiota-depleted mice received microbiota transplantations from donor mice fed control diet; GMT(Cd), mice with gut microbiota depletion received GMT from CdCl_2_-treated mice; GMT (Mel + Cd), mice with microbial depletion received GMT from Mel + Cd mice; GRP78, glucose-regulated protein 78; GPx1, glutathione peroxidase 1; IRE1, inositol-requiring enzyme 1; LC3, microtubule-associated protein light chain 3; Mel, melatonin; NF-κB, nuclear factor kappa B (p65); PINK1, PTEN-induced putative kinase 1; PERK, protein kinase R-like endoplasmic reticulum kinase; PPARα, peroxisome proliferator-activated receptor α; SOD2, superoxide dismutase2; SREBP-1c, Sterol regulatory element-binding protein-1c; TLR, toll-like receptor; TNF-α, tumor necrosis factor α; VDAC, voltage-dependent anion channel; ZO-1, zonula occludens-1. The western blot image is on the left of each bar chart panel. The protein expression value = densitometry units of selected protein/densitometry units of β-actin or VDAC detected by western blotting. β-actin or VDAC was the reference protein to normalize the result of target proteins. Data are expressed as means ± standard errors and presented in the vertical bars (n = 12). Labeled means without a common letter differ (*p* < 0.05).

**Figure 6:**
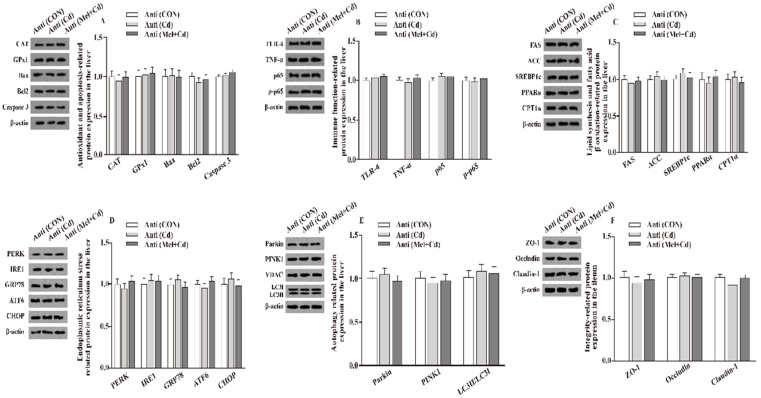
Impact of Mel and CdCl_2_ oral gavage on the abundance of proteins in liver and ileum in antibiotics-treated mice for 10 weeks. The expression levels of relative proteins in antioxidation and apoptosis (**A**), immune function (**B**), fatty acid oxidation and lipid metabolism (**C**), endoplasmic reticulum stress (**D**), autophagy (**E**) in the liver, and integrity (**F**) in the ileum. Anti, antibiotics; Anti (CON), mice with gut microbiota depletion; Anti (Cd), mice with gut microbiota depletion were treated with CdCl_2_; Anti (Mel + Cd), mice with gut microbiota depletion were treated with CdCl_2_ and Mel; ACC, Acetyl-CoA carboxylase; ATF6, activating transcription factor 6; Bcl-2, B-cell lymphoma/leukaemia 2; Bax, Bcl-2-associated X protein; CPT1α, carnitine palmitoyl transferase 1α; CAT, catalase; CHOP, CCAAT/enhancer-binding protein homologous protein; CdCl_2_, cadmium chloride; FAS, fatty acid synthase; GRP78, glucose-regulated protein 78; GPx1, glutathione peroxidase 1; IRE1, inositol-requiring enzyme 1; LC3, microtubule-associated protein light chain 3; Mel, melatonin; NF-κB, nuclear factor kappa B (p65); PINK1, PTEN-induced putative kinase 1; PERK, protein kinase R-like endoplasmic reticulum kinase; PPARα, peroxisome proliferator-activated receptor α; SOD2, superoxide dismutase2; SREBP-1c, Sterol regulatory element-binding protein-1c; TLR, toll-like receptor; TNF-α, tumor necrosis factor α; VDAC, voltage-dependent anion channel; ZO-1, zonula occludens-1. The western blot image is on the left of each bar chart panel. The protein expression value = densitometry units of selected protein/densitometry units of β-actin or VDAC detected by western blotting. β-actin or VDAC was the reference protein to normalize the result of target proteins. Data are expressed as means ± standard errors and presented in the vertical bars (n = 12).

Relative to mice subjected to the Cd and GMT (Cd) treatments, the levels of Caspase 3, Bax, Parkin, LC3II/LC3I, p65, p-p65, TLR-4, FAS, ACC, TNF-α, SREBP-1c, PERK, IRE1, GRP78, ATF6 and CHOP proteins in the liver decreased (*p* *<* 0.05), whereas those of GPx1, CPT1α, CAT, PPARα and Bcl2 in the liver, and those of ZO-1, Claudin-1 and Occludin in ileum, increased (*p* *<* 0.05), respectively, in Mel + Cd and GMT (Mel + Cd) groups (Figure 4 and Figure 5). The oral gavage of CdCl_2_ and Mel did not alter (*p* > 0.05) the levels of apoptotic, antioxidant immune, ER stress, autophagy, lipid-metabolism- and fatty-acid-oxidation-associated proteins in the liver, or those of integrity-associated proteins in the ileum of mice subjected to antibiotic treatment (Figure 6).

### 3.8. Gut Microbial Composition in Cd-Exposed Mice

**Figure 7:**
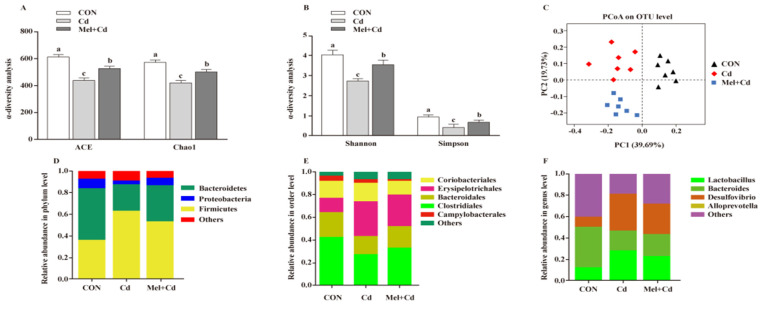
Mel improved gut microbiota of mice exposed to Cd. ACE and Chao1 in ɑ-diversity analysis (**A**), Shannon and Simpson in ɑ-diversity analysis (**B**), PCoA plot study from each sample (**C**), microflora constitutions at the phylum (**D**), order (**E**) and genus (**F**) levels. ACE, abundance-based coverage estimator; CON, mice fed with the control diet; Cd group, mice were given gavage of CdCl_2_ once daily; CdCl_2_, cadmium chloride; Mel + Cd group, mice were treated with Mel and CdCl_2_; Mel, melatonin. Data are expressed as means ± standard errors and presented in the vertical bars (n = 7). Labeled means without a common letter differ (*p <* 0.05).

Mel reversed the Shannon, Simpon, Chao1 and ACE indices caused by Cd (*p* < 0.05) (Figure 7A,B). To further assess the heterogeneous β-diversity, structural alterations of gut microbiota were assessed by PCoA according to the uniFrac distance. As for CON-, Cd- and Mel + Cd-treated mice, significant clustering was observed in terms of microbial composition (Figure 7C). At the phylum level, the relative abundance of Bacteroidetes increased (*p* < 0.05), whereas that of Firmicutes decreased (*p* < 0.05) in mice subjected to the CdCl_2_ and Mel treatments (Figure 7D). At the order level, Cd was found to increase the relative abundance of Erysipelotrichales while decreasing those of Clostridiales and Bacteroidales, and the above alterations were reversed upon Mel exposure (*p* < 0.05) (Figure 7E). Additionally, Mel decreased the relative abundances of Desulfovibrio and Lactobacillus at the genus level (*p* < 0.05) (Figure 7F).

### 3.9. Gut Microbiota Response to GMT

**Figure 8:**
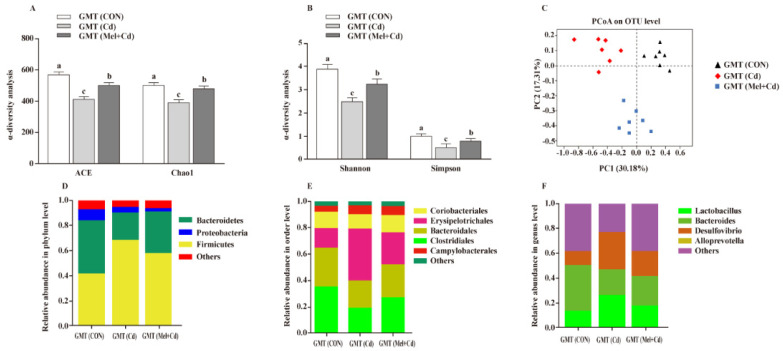
Intestinal microflora responded to microflora transplantation from CON (GMT (CON)), Cd (GMT(Cd)) and Mel + Cd (GMT (Mel + Cd)) group. ACE and Chao1 in ɑ-diversity analysis (**A**), Shannon and Simpson in ɑ-diversity analysis (**B**), PCoA plot study from each sample (**C**), microflora constitutions at the phylum (**D**), order (**E**) and genus (**F**) levels. ACE, abundance-based coverage estimator; CdCl_2_, cadmium chloride; GMT, gut microbiota transplantation; GMT(CON), the microbiota-depleted mice received microbiota transplantations from donor mice fed control diet; GMT(Cd), mice with gut microbiota depletion received GMT from CdCl_2_-treated mice; GMT (Mel + Cd), mice with microbial depletion received GMT from Mel + Cd mice; Mel, melatonin. Data are expressed as means ± standard errors and presented in the vertical bars (n = 7). Labeled means without a common letter differ (*p <* 0.05).

Relative to GMT (Cd)-treated mice, GMT (Mel + Cd) treatment reversed the reduced Shannon, Simpon, Chao1 and ACE indices (*p* < 0.05) (Figure 8A,B). Meanwhile, as detected by PCoA, mice in the GMT (CON), GMT (Cd) and GMT (Mel + Cd) groups showed significant clustering in terms of their microbial composition (Figure 8C). At the phylum level, GMT (Mel + Cd) treatment increased (*p* < 0.05) the relative abundance of Bacteroidetes, whereas it decreased (*p* < 0.05) that of Firmicutes relative to GMT (Cd)-exposed mice (Figure 8D). At the order level, GMT (Mel + Cd) treatment decreased (*p* < 0.05) the relative abundance of Erysipelotrichales while increasing (*p* < 0.05) that of Clostridiales relative to GMT (Cd)-exposed mice (Figure 8E). At the genus level, the relative abundances of Desulfovibrio and Lactobacillus decreased (*p* < 0.05), while that of Bacteroides increased (*p* < 0.05) in mice treated with GMT (Mel + Cd) compared to mice treated with GMT (Cd) (Figure 8F).

### 3.10. Gut Microbial Composition in Mice Treated with Antibiotics

**Figure 9:**
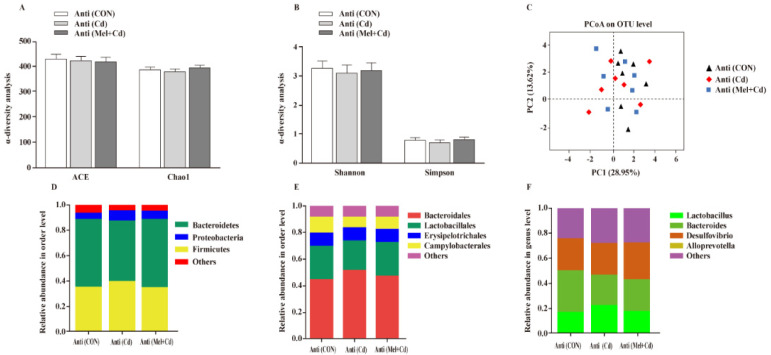
Mel and Cd have little effect on microbiome composition in the colon of antibiotics-treated mice. ACE and Chao1 in ɑ-diversity analysis (**A**), Shannon and Simpson in ɑ-diversity analysis (**B**), PCoA plot study from each sample (**C**), microflora constitutions at the phylum (**D**), order (**E**) and genus (**F**) levels. Anti, antibiotics; Anti (CON), mice with gut microbiota depletion; Anti (Cd), mice with gut microbiota depletion were treated with CdCl_2_; Anti (Mel + Cd), mice with gut microbiota depletion were treated with CdCl_2_ and Mel; ACE, abundance-based coverage estimator; CdCl_2_, cadmium chloride; Mel, melatonin. Data are expressed as means ± standard errors and presented in the vertical bars (n = 7).

According to our PCoA results, the three groups showed similar colonic microbial compositions and diversities (Figure 9A–C), which suggested that Cd and Mel did not affect (*p* > 0.05) gut microbiota among antibiotic-exposed mice. At the phylum order and genus level, neither Cd nor Mel made any difference (*p* > 0.05) in the compositions of colonic microbiota (Figure 9D–F).

## 4. Discussion

Cadmium in the soil or water is recognized to be a significant risk factor for hepatic dysfunction [4]. As revealed by our findings, liver functionality indices such as cell survival and death signaling, the biosynthesis and oxidation of fatty acids, and the oxidative phosphorylation of mitochondria were changed upon environmental Cd exposure in the mouse model. Such hepatic metabolic changes were related to the increased lipid production (an indication of fat accumulation) and the changes in gene expression indicating an interrupted cell cycle, inflammation and subsequent damage to hepatocytes. Such aberrant features in our mouse model indicated that chronic Cd exposure resulted in liver injury.

Interestingly, the serum contents of TG, GLU, CHO and LDL were increased in mice exposed to Cd. The increased contents of TG, GLU and CHO are related to liver function, which possibly facilitates the disordered lipid homeostasis [28,29]. Chronic exposure to Cd changed the above parameters in mice, which indicated the severe impairment of the hepatic lipid metabolism. In the process of lipid metabolism, SREBP-1c regulates the expression of many genes (such as ACC, FAS and SCD1) that code for enzymes involved in fatty acid and lipid biosynthesis [30]. PPARα can modulate different enzymes related to the oxidation of fatty acids [31]. The main function of CPT1α is to control fatty acid flux via β-oxidation, which represents an important regulatory link in the oxidation of fatty acids [32]. As revealed by our results, genes related to biosynthesis (e.g., SREBP1c, PPARγ, FAS, ACC, SCD1) and the oxidation (e.g., PPARα and CPT1α) of fatty acids were significantly up-regulated and down-regulated, respectively, following Cd exposure. These changes were reversed by Mel administration; Mel mitigated Cd-mediated lipid metabolic disorders in the liver by suppressing fatty acid synthesis and enhancing mitochondrial fatty acid oxidation. For verifying whether gut microbiota has been modulated by Mel and if this change is within the role of Mel to ameliorate the Cd-mediated disturbed lipid metabolism in the liver, microbiota-depleted mice underwent GMT from mice given an oral gavage of CON, Cd and Mel + Cd, as well as from mice treated with Cd and Mel after antibiotic treatment. GMT is an important tool to investigate the involvement of gut microbiota in the host immune system under similar genetic backgrounds [33]. In our results, serum GLU, CHO, TG, LDL levels, genes related to liver fatty acid synthesis, and protein expression (PPARγ, SREBP1c, FAS, SCD1, and ACC) were lower, and the mRNA and protein levels of genes related to mitochondrial fatty acid β oxidation (PPARα and CPT1α) were greater in GMT (Mel + Cd) mice compared with GMT(Cd)-treated mice. Oral gavage CdCl_2_ and Mel did not modify serum GLU, CHO, TG and LDL levels, or the expression of liver fatty acid synthesis and mitochondrial fatty acid oxidation in mice subjected to antibiotic treatment. Therefore, the Cd-induced systemic lipid metabolic disorders were possibly mitigated through Mel supplementation by the involvement of gut microbiota.

The mitochondria dysfunctions suppress the β-oxidation of mitochondria and increase the production of the toxic lipid metabolism, which further adversely affects the mitochondrial function and eventually creates a vicious cycle [34]. Mitophagy accounts for the mitochondrial degradation mechanism taking place in the presence of stressful conditions, which is extensively detected in several disorders induced by mitochondrial dysfunction [35]. In this study, it was found that mitophagy activation might be the secondary response to Cd-mediated damage to mitochondria. Therefore, the functional impairment of mitochondria possibly aggravated mitophagy, thereby inducing additional mitochondrial losses. Such a vicious cycle may further develop till the cells die. Mitophagy is detected as a cell death form regardless of whether caspases are activated or not [36]. In this study, exposure to environmental Cd was demonstrated to induce severe liver mitochondrial dysfunction and damage. This mitochondrial dysfunction and damage was indicated by the activated mitophagy (ULK1, ATG5, Beclin-1, LC3-I, LC3-II, PINK1 and Parkin), increased ROS production, hepatic inflammatory response (TNF-ɑ, IL-1β and IL-6) and cell death (apoptotic cell percentage, Bax, Caspase 3), and the decreased hepatic ATP levels, NAD^+^/NADH, complex (I–IV) activities, ΔΨm, and oxidation resistance (GSH-Px, SOD, T-AOC). In contrast, Mel supplementation reversed the above-mentioned mitochondrial damage and dysfunction indicators in the liver. These findings shed more light on the mechanism of Mel supplementation in reversing the Cd-induced inhibition of ATP contents, suppression of respiratory chain reaction and promotion of cell autophagy [37]. In our study, GMT from CON, Cd and Mel + Cd mice showed a similar effect on mitochondrial ROS generation, ΔΨm alterations, mitochondrial complex activities, mitophagy, ATP and apoptosis levels among different groups. Notably, Mel and Cd made no change in liver mitochondrial ROS generation, ΔΨm changes, mitophagy, mitochondrial complex activities, ATP production and apoptosis levels of mice subjected to antibiotic treatment. Therefore, Mel mitigated Cd-induced liver mitochondrial dysfunction in mice by gut microbiota involvement.

This serious and long-time injury is harmful to hepatocytes, which could cause ER stress-associated apoptosis [38]. As revealed by our results, a chronic oral gavage of CdCl_2_ induced severe liver ER stress, while hepatocytes were unable to adapt to these adverse conditions; as a result, they were unable to keep the normal homeostasis of misfolded or unfolded proteins, thereby giving rise to apoptosis and inflammation in hepatocytes. It has been indicated that ER stress exerts an important role in apoptosis and the aberrant lipid metabolism [39]. It was observed in our study that the protein levels of critical liver markers (such as PERK, GRP78, p-IRE1 and ATF6) of unfolded protein response (UPR) pathways were increased in chronic Cd-exposed mice compared with the CON ones. This may be attributed to the fact that ER stress induces the dissociation of GRP78 from transmembrane proteins PERK, IRE1 and ATF6, thereby giving rise to the activation of several downstream apoptotic pathways associated with CHOP and caspase 3, finally giving rise to hepatocyte death [40]. Previous research in nonruminants suggests the involvement of ER stress during lipogenesis [41], and ER stress drives hepatic lipogenesis via SREBP1 [42], which is consistent with our findings. Interestingly, it was also found in our study that Mel was able to eliminate UPR, which suggested the mitigating effect of Mel on Cd-mediated ER stress. In addition, relative to mice subjected to the GMT (Cd) treatment, those subjected to the GMT (Mel + Cd) treatment saw decreased ER stress-associated indicators in the liver. In contrast, the oral gavage of CdCl_2_ and Mel did not affect the ER-stress-associated indicators in the liver of mice under antibiotic treatment. Our results, therefore, showed that Mel mitigated the Cd-induced liver ER stress by gut microbiota involvement.

Disruption of the intestinal barrier function results in changes in intestinal permeability; Cd exposure has been suggested to down-regulate genes related to enhanced intestinal permeability in the mouse gut, such as tight junction (TJ) genes claudin-1 and occludin [43]. The disruption of the intestinal barrier function and the increase in intestinal permeability promotes the translocation of toxic luminal substances and pathogenic microorganisms from the intestine into circulation, and this is related to liver injury development [44]. According to our results, the expression levels of ZO-1, claudin-1 and occludin were up-regulated in the Mel group. Therefore, it was speculated that Mel supplementation promoted the intestinal barrier function for preventing harmful intraluminal penetration, such as by foreign microorganisms and the corresponding toxins [45]. It has been suggested that the function of LPS, bile acids and SCFA is the bridge that links the gut microbiome with liver functions [46]. According to our results, the ileal LPS contents were increased after Cd treatment, which was related to the change in gut microbial structures. The Desulfovibrio-generated endotoxin shows great biological activities, which can induce serious inflammatory responses [47]. It was discovered that Mel decreased the relative abundances of endotoxin-producing bacteria, such as Desulfovibrio, in mice treated with Cd. Butyrate, one of the main SCFA components, plays a vital part in strengthening the intestinal barrier [16]. Clostridiales represent the important bacterium producing butyric acid, which also has an important role in maintaining intestinal homoeostasis [48]. According to our results, mice in the Mel + Cd group increased the relative abundance of Clostridiales that produced butyrate. Therefore, the gut microbiota and corresponding metabolites were possibly the new targets used in the treatment of Cd-induced liver injury. In addition, liver inflammatory cytokines, including IL-1β and TNF-α, can alter LDL and TC contents in Cd-treated rats [29]. In our study, the levels of LPS and inflammatory cytokines in the liver were decreased in the Mel + Cd mice. Consequently, Mel might alleviate Cd-mediated liver inflammation by changing the gut microbiome, which indirectly affects liver functions. It should be noted that Mel and Cd made no change in the intestinal barrier in mice subjected to antibiotic treatment. To better understand the association between Mel-triggered microbial changes and anti-Cd-induced gut barrier injury, microbiota from the CON, Cd and Mel + Cd groups were transplanted into intestinal-microbiota-depleted mice. Similar effects on the composition of colonic microbiota, colonic LPS, SCFA contents, intestinal permeability and liver inflammation were also observed in CON, Cd and Mel + Cd mice. As a result, these findings suggested that the Mel-induced positive modulation of gut microbiota was associated with enhancing liver health and intestinal permeability.

## 5. Conclusions

**Figure 10:**
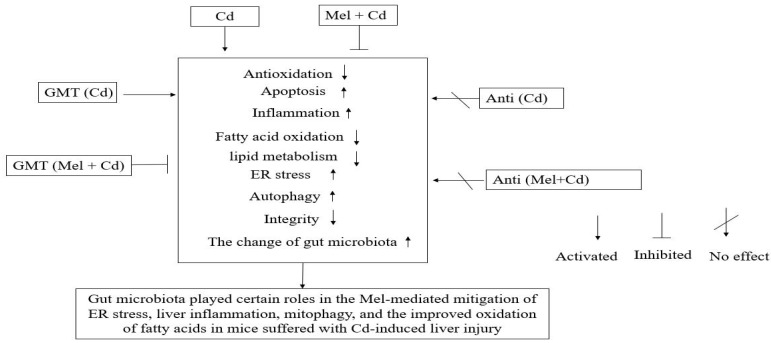
A graphic summary of Mel efficacy in alleviating Cd-induced liver injury involving the gut microbiota and barrier function. Anti, antibiotics; Anti (Cd), mice with gut microbiota depletion were treated with CdCl_2_; Anti (Mel + Cd), mice with gut microbiota depletion were treated with CdCl_2_ and Mel; Cd group, mice given gavage of CdCl_2_ once daily; CdCl_2_, cadmium chloride; ER, endoplasmic reticulum; GMT, gut microbiota transplantation; GMT(Cd), mice with gut microbiota depletion received GMT from CdCl_2_-treated mice; GMT (Mel + Cd), mice with microbial depletion received GMT from Mel + Cd mice; Mel, melatonin; Mel + Cd group, mice given Mel and CdCl_2_ treatment.

A new mechanism is illustrated in this study which links gut microbiota with the effects of Mel on mitigating ER stress, liver inflammation and mitophagy while improving mitochondrial fatty acid oxidation in mice suffering from Cd-induced liver injury (Figure 10). Mel can change the compositions and diversities of colonic microbiota, leading to a reduced production of LPS generated by Gram-negative bacteria, which may enter the liver and blood circulation through intestinal barrier defects. Such impacts are beneficial for alleviating liver inflammation, ER stress and mitophagy, and for improving mitochondrial fatty acid oxidation in mice suffering from Cd-induced liver injury. 

## Data Availability

All data relevant to the study are included in the article or uploaded as supplementary information. Data are available on reasonable request. Data generated and analyzed during this study are available from the corresponding author on reasonable request.

## Supplementary Materials

The following supporting information can be downloaded at: https://www.mdpi.com/article/10.3390/antiox11091727/s1, Table S1: Primer sequences used in the real-time PCR; Table S2: Details of antibodies used for western blotting; Table S3: Effect of Mel supplementation on the growth performance, serum lipid metabolism indices, antioxidative capacity, LPS and cytokine concentrations in the liver in CdCl_2_ treated mice for 10 weeks; Table S4: Effects of GMT from donor mice fed CON, Cd, and Mel + Cd on the growth performance, serum lipid metabolism indices, antioxidative capacity, LPS and cytokine concentrations in the liver in antibiotics-treated mice for 10 weeks; Table S5: Effects of Mel and CdCl_2_ oral gavage on the growth performance, serum lipid metabolism indices, antioxidative capacity, LPS and cytokine concentrations in the liver in antibiotics-treated mice for 10 weeks; Table S6: Effects of Mel supplementation on mitochondrial ROS production, ΔΨm, mitochondrial complex activity, apoptosis levels, NAD^+^/NADH ratio and ATP content in the liver in CdCl_2_ treated mice for 10 weeks; Table S7: Effects of GMT from donor mice fed CON, Cd, and Mel + Cd on the mitochondrial ROS production, ΔΨm, mitochondrial complex activity, apoptosis levels, NAD^+^/NADH ratio and ATP content in the liver in antibiotics-treated mice for 10 weeks; Table S8: Effects of Mel and CdCl_2_ oral gavage on mitochondrial ROS production, ΔΨm, mitochondrial complex activity, apoptosis levels, NAD^+^/NADH ratio and ATP content in the liver in antibiotics-treated mice for 10 weeks; Table S9: Effect of Mel supplementation on the Cd residues in liver and ileum and Mel level in the serum in CdCl_2_ treated mice for 10 weeks; Table S10: Effects of GMT from donor mice fed CON, Cd, and Mel + Cd on the Cd residues in liver and ileum and Mel level in the serum in antibiotics-treated mice for 10 weeks; Table S11: Effects of Mel and CdCl_2_ oral gavage on the Cd residues in liver and ileum and Mel level in the serum in antibiotics-treated mice for 10 weeks; Table S12: Effect of Mel supplementation on the villus morphology, barrier function, anti-oxidative capacity, LPS and cytokine concentrations in the ileum, and DAO activity and D-lactic acid concentration in the serum in CdCl_2_ treated mice for 10 weeks; Table S13: Effects of GMT from donor mice fed CON, Cd, and Mel + Cd on the villus morphology, barrier function, anti-oxidative capacity, LPS and cytokine concentrations in the ileum, and DAO activity and D-lactic acid concentration in the serum in antibiotics-treated mice for 10 weeks; Table S14: Effects of Mel and CdCl_2_ oral gavage on the villus morphology, barrier function, anti-oxidative capacity, LPS and cytokine concentrations in the ileum, and DAO activity and D-lactic acid concentration in the serum in antibiotics-treated mice for 10 weeks; Table S15: Effects of Mel supplementation on SCFAs and LPS concentrations in colonic contents in CdCl_2_ treated mice for 10 weeks; Table S16: Effects of GMT from donor mice fed CON, Cd, and Mel + Cd on SCFAs and LPS concentrations in colonic contents in antibiotics-treated mice for 10 weeks; Table S17: Effects of Mel and CdCl_2_ oral gavage on SCFAs and LPS concentrations in colonic contents in antibiotics-treated mice for 10 weeks.
