# Supplementary material for: Effects of the Gut Microbiota and Barrier Function on Melatonin Efficacy in Alleviating Liver Injury
Source: Antioxidants (Basel). 2022 Aug 31;11(9):1727. doi: 10.3390/antiox11091727 (PMC9495757; doi:10.3390/antiox11091727)
Supplement: Supplementary file 1 [file antioxidants-11-01727-s001.zip › antioxidants-1880140-supplementary.pdf]

Table S1 Primer sequences used in the real-time PCR

Gene	Sequences (5′–3′)	GenBank accession number
CAT	F: CCTCGTTCAGGATGTGGT TT	NM_009804
	R: TCTGGTGATATCGTGGGTGA	
GPx1	F: CCTCAAGTACGTCCGACCTG	NM_001329528.1
	R: CAATGTCGTTGCGGCACACC	
SOD2	F: GGCTGGCTTGGCTTCAATAA	NM_013671.3
	R: GCGGAATAAGGCCTGTTGTT	
P53	F: TACAAGAAGTCACAGCACAT	AB020317.1
	R: GATAGGTCGGCGGTTCAT	
Bax	F: TGGAGATGAACTGGACAGCAATAT	NM_007527. 3
	R: GCAAAGTAGAAGAGGGCAACCAC	
Bcl2	F: CATTATCAATGATGTACCATG	NM_009741.3
	R: GCAGTAAATAGCTGATTCGAC	
Caspase 3	F: CAAAGCGCAGTGTCCTGCGG	NM_009810.3
	R: ACCCCGGCAGGCCTGAATGA	
TLR-4	F: TTTGCTGGGGCTCATTCACT	NM_021297.3
	R: GACTCGGCACTTAGCACTGT	
IL-1β	F: CCTGGGCTGTCCTGATGAGAG	NM_008361.4
	R: TCCACGGGAAAGACACAGGTA	
NF-κB	F: GGCCCTAAAGATTGTGCCAAG	NM_008689.2
	R: AGCAACATCTTCACATCCCCC	
TNF-α	F: TGGGACAGTGACCTGGACTGT	NM_013693.2
	R: TTCGGAAAGCCCATTTGAGT	
PERK	F：CACGCAGATCACAGTCAGGT	NM_010121.3
	R：GGGCTGAGGATGGAAAAGCC	
IRE1	F: TTTAGCTTTGCCGACCGTGA	NM_023913.2
	R: TGCACACAGCTCGATAGCAA	

GRP78	F: ATCGTGCCTCTCATTGGTGG	U16277.1
	R: TAGTTGGAGGCCGCTGATTG	
ATF6	F: ATGGGTTCGGATATCGCTGT	NM_001081304.1
	R: TTCTTCTTCTTGCGCGACTG	
CHOP	F: AGTGCATCTTCATACACCACCACA	NM_007837.4
	R: CAGATCCTCATACCAGGCTTCCA	
FAS	F：CCTCGTGATGAACGTGTACC	NM_007988.3
	R：TGAGGACGTTTACAAAGGCA	
ACC	F: GCCAGTGCTATGCTGAGATTGAG	NM_133360.2
	R: CTATCACACAGCCAGGGTCAAGT	
CD36	F: GAACCTATTGAAGGCTT ACATCC	NM_001159557.1
	R: CCCA GTCACTTGTGTTTTGAAC	
CPT1α	F: GACTCCGCTCGCTCATTCC	NM_013495.2
	R: ACCAGTGATGATGCCATTCTTG	
SREBP-1c	F: AGAGCGAGCGTTGAACTGTATT	NM_011480.3
	R: ATCCAAGGGCATCTGAGAACTC	
PPARα	F: TGCATGTCCGTGGAGACCGTCAC	NM_001113418.1
	R: ACTCGGTCTTCTTGATGACC	
PPARγ	CCTGGCAAAGCATTTGTATG	NM_001127330.1
	TGGTGATTTGTCCGTTGTCT	
SCD-1	F: CTACACCTGCCTCTTCGGGATT	NM_009127.4
	R: CCGTGCCTTGTAAGTTCTGTG	
ULK1	F: AGTTCTGGAGATTGCAGCCC	NM_009469.3
	R: ACCACACTTTCCTGGAGCTG	
LC3	F: CCTGGACAAGACCAAGTTCC	NM_026160.4
	R: GTCTCCTGCGAGGCATAAAC	
ATG5	F: TTGAGTCAGGACAACGAGGC	NM_001314013.1
	R: TTCTCCTCCTTGGCTTGCAG	

Beclin1	F: GAAACTGGACACGAGCTTCAAGA<br>R: ACCATCCTGGCGAGTTTCAATA	NM_001034117.1
PINK1	F: GCTTTGGCTGGAGAGTATGG<br>R: CGGATGATGTTAGGGTGTGG	NM_026880.2
Parkin	F: ACACAGACAGTAAGAGGGATTCAGAA<br>R: GTGAGGGTTGCTTGTTTGCA	NM_001317726.1
ZO-1	F: ATGGAAAGCTGGGCTCTTGGCT<br>R: ACCACCCGCTGTCTTTGGAAGT	D14340.1
Occludin	F: TTCAGGTGAATGGGTCACCG<br>R: AGATAAGCGAACCTGCCGAG	NM_001360536.1
Claudin-1	F: AGACCTGGATTTGCATCTTGGTG<br>R: TGCAACATAGGCAGGACAAGAGTTA	NM_016674.4
β-actin	F: AGCCATGTACGTAGCCATCC<br>R: CTCTCAGCTGTGGTGGTGAA	NM007393

CAT, catalase; GPx1, glutathione peroxidase 1; SOD2, superoxide dismutase 2; Bax, Bcl-2-associated X protein; Bcl-2, B-cell lymphoma/leukaemia 2; TLR, toll-like receptor; IL, interleukin, TNF-α, tumor necrosis factor α; NF-κB, nuclear factor kappa B (p65); PERK, protein kinase R-like endoplasmic reticulum kinase; IRE1, inositol-requiring enzyme 1; GRP78, glucose-regulated protein 78; ATF6, activating transcription factor 6; CHOP, CCAAT/enhancer-binding protein homologous protein; FAS, fatty acid synthase; ACC, Acetyl-CoA carboxylase; CPT1α, carnitine palmitoyltransferase 1α; SREBP-1c, Sterol regulatory element binding protein-1c; PPARα, peroxisome proliferator-activated receptor α; PPARγ, Peroxisome proliferator-activated receptor gamma; SCD-1, stearoyl-CoA desaturase-1; ZO-1, zonula occludens-1; LC3, microtubule-associated protein light chain 3; ULK1, unc-51 like autophagy activating kinase 1; PINK1, PTEN induced putative kinase 1; ATG5, autophagy related 5; F, forward; R, reverse.

Table S2 Details of antibodies used for western blotting in this study

Antibodies	Cat NO.	Source	Dilutions of Western blot
anti-GPx1	ab59546	Abcam (MA, USA)	1:1000
anti-CAT	20225-1-AP	Protein Tech (CHI, USA)	1:1000
anti-P53	11897-1-AP	Protein Tech (CHI, USA)	1:1000
anti-SOD2	NB100-1992	Novus (CO, USA)	1:2000
anti-Bcl2	Ab56361	Abcam (MA, USA)	1:1000
anti-CHOP	CS19116	Cell Signaling (MA, USA)	1:1000
anti-Caspase 3	CS16843	Cell Signaling (MA, USA)	1:1000
anti-Bax	CS20819	Cell Signaling (MA, USA)	1:1000
anti-p65	CS20976	Cell Signaling (MA, USA)	1:500
anti-pp65	CS19807	Cell Signaling (MA, USA)	1:500
anti-TNF-α	SC-511	Santa Cruz Biotechnology (CA, USA)	1:500
anti-TLR4	Ab40975	Abcam (MA, USA)	1:1000
anti-PERK	C33E18	Cell Signaling (MA, USA)	1:1000
anti-IRE1	ab37073	Abcam (MA, USA)	1:1000
anti-ATF6	ab03119	Abcam (MA, USA)	1:1000
anti-GRP78	SC-376768	Santa Cruz Biotechnology (CA, USA)	1:250
FAS	3180	Cell Signaling (MA, USA)	1:1000
ACC	3662	Cell Signaling (MA, USA)	1:1000
CPT1α	ab53532	Abcam (MA, USA)	1:500
PPARα	SC-130640	Santa Cruz Biotechnology (CA, USA)	1:1000
SREBP1c	NB100-2215	Novus (CO, USA)	1:1000
anti-ZO-1	SC-487	Santa Cruz Biotechnology (CA, USA)	1:1000
anti-claudin-1	SC-319	Santa Cruz Biotechnology (CA, USA)	1:1000
anti-occludin	SC-503	Santa Cruz Biotechnology (CA, USA)	1:1000
anti-LC3	SC-489	Santa Cruz Biotechnology (CA, USA)	1:1500

anti-Parkin	SC-726	Santa Cruz Biotechnology (CA, USA)	1:1000
anti-PINK1	SC-719	Santa Cruz Biotechnology (CA, USA)	1:1000
anti-*β*-actin	SC-820	Santa Cruz Biotechnology (CA, USA)	1:1000
anti-VDAC	SC-513	Santa Cruz Biotechnology (CA, USA)	1:1500
HRP-labelled goat anti-rabbit IgG (secondary antibodies)	AB-98328	Antgene Biotech (Wuhan, China)	1:5000

CAT, catalase; GPx1, glutathione peroxidase 1; SOD2, superoxide dismutase2; Bax, Bcl-2-associated X protein; Bcl-2, B-cell lymphoma/leukaemia 2; CHOP, CCAAT/enhancer-binding protein homologous protein; PERK, protein kinase R-like endoplasmic reticulum kinase; IRE1, inositol-requiring enzyme 1; GRP78, glucose-regulated protein 78; ATF6, activating transcription factor 6; FAS, fatty acid synthase; ACC, Acetyl-CoA carboxylase; CPT1α, carnitine palmitoyl transferase 1α; SREBP-1c, Sterol regulatory element binding protein-1c; PPARα, peroxisome proliferator-activated receptor α; TLR, toll-like receptor; TNF-α, tumor necrosis factor α; NF-κB, nuclear factor kappa B (p65); ZO-1, zonula occludens-1; LC3, microtubule-associated protein light chain 3; PINK1, PTEN induced putative kinase 1; HRP, horseradish peroxidase; VDAC, voltage dependent anion channel.

Table S3 Effect of melatonin supplementation on the growth performance, serum lipid metabolism indices, antioxidative capacity, LPS and cytokine concentrations in the liver in $CdCl_2$ treated mice for 10 weeks [a].

Item[b]	CON	Cd	Mel+Cd	SEM	P value
**Growth performance**					
Initial weight, g	21.20	21.25	21.15	0.83	0.135
Final weight, g	31.91[a]	23.67[c]	27.92[b]	1.04	0.023
Body weight gain, g	10.71[a]	2.42[c]	6.77[b]	0.79	0.009
Liver weight, g	0.97 [c]	1.28[a]	1.16[b]	0.08	0.012
Liver index, %	3.04 [c]	5.39[a]	4.15[b]	0.49	0.007
**Serum enzyme activities and Lipid metabolism indices**					
Serum ALB, g/L	46.16[a]	30.33[c]	39.18[b]	2.99	0.013
Serum ALP, U/L	54.18[c]	101.21[a]	69.21[b]	3.12	0.007
Serum TG, mM	0.62[c]	1.34[a]	0.86[b]	0.08	0.031
Serum TCHO, mM	3.02[c]	5.69[a]	4.18[b]	0.58	0.007
Serum HDL, mM	2.13	2.08	2.19	0.37	0.231
Serum LDL, mM	0.83[c]	1.39[a]	1.05[b]	0.07	0.006
Serum glucose , mM	3.12[c]	6.98[a]	4.68[b]	0.47	0.019
Serum insulin, μU/mL	8.18[c]	15.27[a]	12.82[b]	1.02	0.008
HOMA-IR	1.13[c]	4.74[a]	2.67[b]	0.27	0.013
Liver CPT1 activity , μmol/min/mg	5.43[a]	2.78[c]	3.69[b]	0.39	0.009
Liver TG, μmol/g	6.93[c]	13.28[a]	9.19[b]	0.41	0.014
**Antioxidative capacity**					

GSH-Px, U/mg prot	40.12^{a}	22.47^{c}	31.35^{b}	1.37	0.028
SOD, U/mg prot	54.27^{a}	32.49^{c}	43.21^{b}	1.89	0.012
T-AOC, U/mg prot	5.12^{a}	3.67^{b}	4.89^{ab}	0.15	0.039
MDA, nmol/mg prot	1.31^{c}	2.16^{a}	1.64^{b}	0.09	0.007
**Liver LPS and cytokine concentrations**					
LPS, ng/mg prot	2.98^{c}	6.02^{a}	4.28^{b}	0.11	0.005
TNF-α, ng/mg prot	8.09^{c}	13.48^{a}	10.29^{b}	0.86	0.019
IL-1β, ng/mg prot	11.28	11.98	11.69	0.79	0.097
IL-6, ng/mg prot	13.26^{c}	21.89^{a}	16.97^{b}	1.04	0.008

[a] Mean value with their standard errors of the mean (SEM) (n = 12 per group). Within a row, mean values without a common letter differ ($P < 0.05$).

[b] CON, mice in the control group fed a standard lab diet; Cd group, mice were challenged with $CdCl_2$ once a day by gavage; Mel+Cd group, the $CdCl_2$ mice were treated with melatonin; TG, triglyceride; HDL, high-density lipoprotein; LDL, low-density lipoprotein; TCHO, total cholesterol; CPT1, carnitine palmitoyl transferase 1; GSH-Px, glutathione peroxidase; SOD, superoxide dismutase; T-AOC, total antioxidant capacity; MDA, malondialdehyde; LPS, lipopolysaccharide; TNF-α, tumor necrosis factor α; IL, interleukin; ALB, albumin; ALP, alkaline phosphatase; HOMA-IR, homeostasis model assessment-insulin resistance; HOMA-IR is calculated as serum glucose (mM)× serum insulin (μU/mL)/22.5; liver index is calculated as liver weight/bodyweight (week 10) × 100%.

Table S4 Effects of gut microbiota transplantation from donor mice fed CON, Cd, and Mel +Cd on the growth performance, serum lipid metabolism indices, antioxidative capacity, LPS and cytokine concentrations in the liver in antibiotics-treated mice for 10 weeks [a].

Item[b]	GMT (CON)	GMT (Cd)	GMT (Mel+Cd)	SEM	P value
**Growth performance**					
Initial weight, g	21.30	21.34	21.32	0.79	0.218
Final weight, g	27.62[a]	23.23[b]	25.39[ab]	1.56	0.036
Body weight gain, g	6.32[a]	1.89[c]	4.07[b]	0.24	0.011
Liver weight, g	0.84 [c]	1.23[a]	1.06[b]	0.06	0.022
Liver index, %	3.01[c]	5.29[a]	4.18[b]	0.33	0.009
**Serum enzyme activities and Lipid metabolism indices**					
Serum ALB, g/L	40.23[a]	24.16[c]	32.57[b]	3.04	0.008
Serum ALP, U/L	42.34[c]	89.43[a]	60.16[b]	2.98	0.011
Serum TG, mM	0.52[c]	1.12[a]	0.79[b]	0.06	0.015
Serum TCHO, mM	2.63	5.14[a]	3.89[b]	0.47	0.009
Serum HDL, mM	2.09	2.12	2.11	0.31	0.108
Serum LDL, mM	0.72[c]	1.28[a]	0.93[b]	0.05	0.017
Serum glucose, mM	2.99[c]	6.12[a]	4.19[b]	0.29	0.024
Serum insulin, μU/mL	7.85[c]	13.94[a]	10.15[b]	1.59	0.016
HOMA-IR	1.04[c]	3.79[a]	1.89[b]	0.23	0.008
Liver CPT1 activity, μmol/min/mg	4.99[a]	2.19[c]	3.21[b]	0.31	0.018
Liver TG, μmol/g	5.87[c]	10.13[a]	7.75[b]	0.38	0.008
**Antioxidative capacity**					

GSH-Px, U/mg prot	35.58^{a}	19.86^{c}	27.54^{b}	1.36	0.013
SOD, U/mg prot	47.48^{a}	29.79^{c}	36.74^{b}	1.68	0.009
T-AOC, U/mg prot	4.53^{a}	2.28^{c}	3.36^{b}	0.10	0.015
MDA, nmol/mg prot	1.26^{c}	1.99^{a}	1.58^{b}	0.07	0.005
**Liver LPS and cytokine concentrations**					
LPS, ng/mg prot	2.03^{c}	5.11^{a}	3.16^{b}	0.14	0.012
TNF-α, ng/mg prot	6.16^{c}	11.24^{a}	8.15^{b}	0.79	0.007
IL-1β, ng/mg prot	9.16	9.21	9.15	0.644	0.142
IL-6, ng/mg prot	10.14^{c}	18.75^{a}	14.26^{b}	1.12	0.015

[a] Mean value with their standard errors of the mean (SEM) (n = 12 per group). Within a row, mean values without a common letter differ ($P < 0.05$).

[b] GMT, gut microbiota transplantation; GMT(CON), the microbiota-depleted mice that were originally fed control diet received microbiota transplantations from donor mice fed control diet; GMT(Cd), the microbiota-depleted mice that were originally fed control diet received microbiota transplantations from donor mice treated $CdCl_2$; GMT (Mel+Cd), the microbiota-depleted mice that were originally fed control diet received microbiota transplantations from donor mice in Mel + Cd groups; TG, triglyceride; HDL, high-density lipoprotein; LDL, low-density lipoprotein; TCHO, total cholesterol; CPT1, carnitine palmitoyl transferase 1; GSH-Px, glutathione peroxidase; SOD, superoxide dismutase; T-AOC, total antioxidant capacity; MDA, malondialdehyde; LPS, lipopolysaccharide; TNF-α, tumor necrosis factor α; IL, interleukin; ALB, albumin; ALP, alkaline phosphatase; HOMA-IR, homeostasis model assessment-insulin resistance; HOMA-IR is calculated as serum glucose (mM)× serum insulin (μU/mL)/22.5; liver index is calculated as liver weight/bodyweight (week 10) × 100%.

Table S5 Effects of melatonin and Cdcl2 oral gavage on the growth performance, serum lipid metabolism indices, antioxidative capacity, LPS and cytokine concentrations in the liver in antibiotics-treated mice for 10 weeks [a].

Item[b]	Anti (CON)	Anti (Cd)	Anti (Mel+Cd)	SEM	P value
**Growth performance**					
Initial weight, g	21.76	21.80	21.81	0.92	0.208
Final weight, g	23.22	23.25	23.23	1.27	0.104
Body weight gain, g	1.46	1.45	1.42	0.31	0.086
Liver weight, g	0.81	0.80	0.78	0.08	0.212
Liver index, %	3.49	3.44	3.36	0.29	0.152
**Serum enzyme activities**					
**and Lipid metabolism indices**					
Serum ALB, g/L	34.13	32.57	33.97	2.16	0.219
Serum ALP, U/L	38.45	41.42	39.53	1.96	0.117
Serum TG, mM	0.55	0.58	0.56	0.06	0.095
Serum TCHO, mM	2.37	2.39	2.32	0.29	0.205
Serum HDL, mM	1.98	2.01	1.96	0.29	0.103
Serum LDL, mM	0.76	0.80	0.78	0.09	0.116
Serum glucose, mM	3.01	2.98	3.04	0.31	0.214
Serum insulin, μU/mL	7.54	7.58	7.49	1.23	0.092
HOMA-IR	1.01	0.99	1.01	0.16	0.213
Liver CPT1 activity, μmol/min/mg	5.02	4.91	4.99	0.31	0.099
Liver TG, μmol/g	5.53	5.57	5.55	0.36	0.138
**Antioxidative capacity**					
GSH-Px, U/mg prot	31.34	32.08	31.96	1.28	0.106

SOD, U/mg prot	42.67	42.09	42.38	1.63	0.207
T-AOC, U/mg prot	4.19	4.14	4.18	0.11	0.181
MDA, nmol/mg prot	1.12	1.18	1.14	0.07	0.163
**Liver LPS and cytokine concentrations**					
LPS, ng/mg prot	1.95	2.01	1.94	0.16	0.092
TNF-α, ng/mg prot	5.32	5.40	5.37	0.78	0.194
IL-1β, ng/mg prot	9.34	9.43	9.41	0.58	0.115
IL-6, ng/mg prot	9.79	9.86	9.83	1.22	0.224

[a] Mean value with their standard errors of the mean (SEM) (n = 12 per group). Within a row, mean values without a common letter differ ($P < 0.05$).

[b] Anti, antibiotics; Anti (CON), the microbiota-depleted mice that were originally fed control diet; Anti (Cd), the microbiota-depleted mice that were originally fed control diet treated $CdCl_2$; Anti (Mel+Cd), the microbiota-depleted mice that were originally fed control diet treated melatonin and $CdCl_2$; TG, triglyceride; HDL, high-density lipoprotein; LDL, low-density lipoprotein; TCHO, total cholesterol; CPT1, carnitine palmitoyl transferase 1; GSH-Px, glutathione peroxidase; SOD, superoxide dismutase; T-AOC, total antioxidant capacity; MDA, malondialdehyde; LPS, lipopolysaccharide; TNF-α, tumor necrosis factor α; IL, interleukin; ALB, albumin; ALP, alkaline phosphatase; HOMA-IR, homeostasis model assessment-insulin resistance; HOMA-IR is calculated as serum glucose (mM)×serum insulin (μU/mL)/22.5; liver index is calculated as liver weight/bodyweight (week 10) × 100%.

Table S6 Effects of melatonin supplementation on mitochondrial ROS production, ΔΨm, mitochondrial complex activity, apoptosis levels, NAD+/NADH ratio and ATP content in the liver in CdCl2 treated mice for 10 weeks[a].

Item[b]	CON	Cd	Mel+Cd	SEM	P value
ROS production, fold change	1.00[c]	3.04[a]	1.87[b]	0.12	0.009
ΔΨm, fold change	1.00[a]	0.53[c]	0.77[b]	0.09	0.012
Apoptotic cells, %	3.16[c]	9.86[a]	5.63[b]	0.19	0.021
Necrotic cells, %	0.45[c]	0.89[a]	0.68[b]	0.07	0.010
NAD^+/NADH	4.47[a]	2.09[c]	3.31[b]	0.18	0.009
ATP content, fold change	1.00[a]	0.48[c]	0.69[b]	0.09	0.007
Complex I, nmol/min/mg protein	209.37[a]	137.81[c]	165.55[b]	5.97	0.024
Complex II, nmol/min/mg protein	278.11[a]	231.12[b]	251.83[ab]	6.11	0.019
Complex III, nmol/min/mg protein	378.19[a]	295.58[c]	339.73[b]	7.10	0.021
Complex IV, nmol/min/mg protein	254.29[a]	178.19[c]	209.29[b]	4.99	0.009

[a] Mean value with their standard errors of the mean (SEM) (n = 12 per group). Within a row, mean values without a common letter differ ($P < 0.05$).

[b] CON, mice in the control group fed a standard lab diet; Cd group, mice were challenged with $CdCl_2$ once a day by gavage; Mel+Cd group, the $CdCl_2$ mice were treated with melatonin; ROS, reactive oxygen species; ATP, adenosine triphosphate; ΔΨm, mitochondrial membrane potential change; NAD, Nicotinamide adenine dinucleotide.

Table S7 Effects of gut microbiota transplantation from donor mice fed CON, Cd, and Mel +Cd on the mitochondrial ROS production, ΔΨm, mitochondrial complex activity, apoptosis levels, $NAD^+/NADH$ ratio and ATP content in the liver in antibiotics-treated mice for 10 weeks [a].

Item[b]	GMT (CON)	GMT(Cd)	GMT (Mel+Cd)	SEM	P value
ROS production, fold change	1.00[c]	2.54[a]	1.42[b]	0.09	0.009
ΔΨm, fold change	1.00[a]	0.64[c]	0.81[b]	0.07	0.018
Apoptotic cells, %	3.01[c]	7.36[a]	4.85[b]	0.13	0.011
Necrotic cells, %	0.37[c]	0.77[a]	0.56[b]	0.06	0.006
$NAD^+/NADH$	4.06[a]	1.57[c]	2.96[b]	0.12	0.007
ATP content, fold change	1.00[a]	0.52[c]	0.75[b]	0.07	0.009
Complex I, nmol/min/mg protein	192.48[a]	124.25[c]	156.12[b]	3.97	0.011
Complex II, nmol/min/mg protein	256.48[a]	207.89[c]	231.32[b]	5.09	0.028
Complex III, nmol/min/mg protein	346.22[a]	314.89[b]	330.12[ab]	6.38	0.006
Complex IV, nmol/min/mg protein	268.12[a]	201.52[c]	228.12[b]	5.01	0.007

[a] Mean value with their standard errors of the mean (SEM) (n = 12 per group). Within a row, mean values without a common letter differ ($P < 0.05$).

[b] GMT, gut microbiota transplantation; GMT(CON), the microbiota-depleted mice that were originally fed control diet received microbiota transplantations from donor mice fed control diet; GMT(Cd), the microbiota-depleted mice that were originally fed control diet received microbiota transplantations from donor mice treated $CdCl_2$; GMT (Mel+Cd), the microbiota-depleted mice that were originally fed control diet received microbiota transplantations from donor mice in Mel + Cd groups; ROS, reactive oxygen species; ATP, adenosine triphosphate; ΔΨm, mitochondrial membrane potential change; NAD, Nicotinamide adenine dinucleotide.

Table S8 Effects of melatonin and Cdcl2 oral gavage on mitochondrial ROS production, ΔΨm, mitochondrial complex activity, apoptosis levels, NAD^+/NADH ratio and ATP content in the liver in antibiotics-treated mice for 10 weeks [a].

Item[b]	Anti (CON)	Anti (Cd)	Anti (Mel+Cd)	SEM	P value
ROS production, fold change	1.00	1.06	0.98	0.10	0.163
ΔΨm, fold change	1.00	0.96	1.04	0.11	0.142
Apoptotic cells, %	2.98	3.05	3.01	0.10	0.209
Necrotic cells, %	0.32	0.39	0.36	0.08	0.115
NAD^+/NADH	3.89	3.94	3.91	0.14	0.104
ATP content, fold change	1.00	0.89	0.94	0.06	0.099
Complex I, nmol/min/mg protein	189.25	190.13	185.68	5.11	0.216
Complex II, nmol/min/mg protein	239.47	241.58	237.69	4.99	0.117
Complex III, nmol/min/mg protein	357.38	349.12	353.17	5.93	0.089
Complex IV, nmol/min/mg protein	235.19	240.21	237.64	5.06	0.182

[a] Mean value with their standard errors of the mean (SEM) (n = 12 per group). Within a row, mean values without a common letter differ ($P < 0.05$).

[b] Anti, antibiotics; Anti (CON), the microbiota-depleted mice that were originally fed control diet; Anti (Cd), the microbiota-depleted mice that were originally fed control diet treated $CdCl_2$; Anti (Mel+Cd), the microbiota-depleted mice that were originally fed control diet treated melatonin and $CdCl_2$; ROS, reactive oxygen species; ATP, adenosine triphosphate; ΔΨm, mitochondrial membrane potential change; NAD, Nicotinamide adenine dinucleotide.

Table S9 Effect of melatonin supplementation on the Cd residues in liver and ileum and melatonin level in the serum in $CdCl_2$ treated mice for 10 weeks[a].

Item[b]	CON	Cd	Mel+Cd	SEM	P value
Melatonin, pg/mL	22.34[a]	12.10[c]	17.86[b]	1.59	0.007
Cd residues, µg/g wet weight					
Liver	0.047[c]	4.02[a]	2.61[b]	0.12	0.014
Ileum	0.029[c]	2.56[a]	1.32[b]	0.09	0.008

[a] Mean value with their standard errors of the mean (SEM) (n = 12 per group). Within a row, mean values without a common letter differ ($P < 0.05$).

[b] Cd, cadmium; CON, mice in the control group fed a standard lab diet; Cd group, mice were challenged with $CdCl_2$ once a day by gavage; Mel+Cd group, the $CdCl_2$ mice were treated with melatonin.

Table S10 Effects of gut microbiota transplantation from donor mice fed CON, Cd, and Mel +Cd on the Cd residues in liver and ileum and melatonin level in the serum in antibiotics-treated mice for 10 weeks [a].

Item[b]	GMT (CON)	GMT (Cd)	GMT (Mel+Cd)	SEM	P value
Melatonin, pg/mL	19.15[a]	10.67[c]	14.68[b]	1.49	0.017
Cd residues, μg/g wet weight					
Liver	0.034[c]	3.24[a]	1.98[b]	0.08	0.009
Ileum	0.017[c]	1.99[a]	1.01[b]	0.11	0.019

[a] Mean value with their standard errors of the mean (SEM) (n = 12 per group). Within a row, mean values without a common letter differ ($P < 0.05$).

[b] Cd, cadmium; GMT, gut microbiota transplantation; GMT(CON), the microbiota-depleted mice that were originally fed control diet received microbiota transplantations from donor mice fed control diet; GMT(Cd), the microbiota-depleted mice that were originally fed control diet received microbiota transplantations from donor mice treated $CdCl_2$; GMT (Mel+Cd), the microbiota-depleted mice that were originally fed control diet received microbiota transplantations from donor mice in Mel + Cd groups.

Table S11 Effects of melatonin and Cdcl2 oral gavage on the Cd residues in liver and ileum and melatonin level in the serum in antibiotics-treated mice for 10 weeks [a].

Item[b]	Anti (CON)	Anti (Cd)	Anti (Mel+Cd)	SEM	P value
Melatonin, pg/mL	12.05[b]	11.96[b]	18.19[a]	1.37	0.012
Cd residues, μg/g wet weight					
Liver	0.029[b]	3.37[a]	2.32[a]	0.06	0.014
Ileum	0.014[b]	2.16[a]	2.14[a]	0.05	0.026

[a] Mean value with their standard errors of the mean (SEM) (n = 12 per group). Within a row, mean values without a common letter differ ($P < 0.05$).

[b] Cd, cadmium; Anti, antibiotics; Anti (CON), the microbiota-depleted mice that were originally fed control diet; Anti (Cd), the microbiota-depleted mice that were originally fed control diet treated $CdCl_2$; Anti (Mel+Cd), the microbiota-depleted mice that were originally fed control diet treated melatonin and $CdCl_2$.

Table S12 Effect of melatonin supplementation on the villus morphology, barrier function, anti-oxidative capacity, LPS and cytokine concentrations in the ileum, and DAO activity and D-lactic acid concentration in the serum in $CdCl_2$ treated mice for 10 weeks[a].

Item[b]	CON	Cd	Mel+Cd	SEM	P value
**Ileal villus morphology**					
Villous height, μm	289.14[a]	197.17[c]	235.34[b]	8.99	0.007
Crypt depth, μm	110.75[c]	159.61[a]	132.96[b]	4.11	0.021
VCR	2.61[a]	1.24[c]	1.77[b]	0.23	0.009
**Ileal barrier function**					
TER, $\Omega \cdot cm^2$	66.89[a]	41.88[c]	52.14[b]	3.27	0.011
FD4 flux, $\mu g \cdot cm^{-2} \cdot h^{-1}$	2.01[c]	3.68[a]	2.71[b]	0.17	0.009
**Ileal anti-oxidative capacity**					
GSH-Px, U/mg prot	35.89[a]	16.99[c]	25.86[b]	1.58	0.015
SOD, U/mg prot	49.89[a]	27.48[c]	36.53[b]	1.97	0.008
T-AOC, U/mg prot	6.08[a]	3.05[c]	4.96[b]	0.11	0.021
MDA, nmol/mg prot	1.12[c]	1.98[a]	1.49[b]	0.06	0.013
**Ileal LPS and cytokine concentrations**					
LPS, ng/mg prot	3.01[c]	5.99[a]	4.11[b]	0.09	0.007
TNF-α, ng/mg prot	7.11[c]	12.29[a]	9.03[b]	0.69	0.013
IL-1β, ng/mg prot	9.38[b]	13.12[a]	11.07[ab]	0.66	0.028
IL-6, ng/mg prot	11.98[c]	18.75[a]	15.18[b]	0.95	0.019
**Serum**					
DAO, $U\ mL^{-1}$	1.15[c]	1.95[a]	1.61[b]	0.06	0.014

D-lactic acid, mg L^{-1}	0.84^{c}	1.59^{a}	1.15^{b}	0.08	0.007

^{a} Mean value with their standard errors of the mean (SEM) (n = 12 per group). Within a row, mean values without a common letter differ ($P < 0.05$).

^{b} CON, mice in the control group fed a standard lab diet; Cd group, mice were challenged with $CdCl_2$ once a day by gavage; Mel+Cd group, the $CdCl_2$ mice were treated with melatonin; VCR, villous height/crypt depth ratio; DAO, diamine oxidase; TER, transepithelial electrical resistance; FD4, fluorescein isothiocyanate dextran (4 kDa); TNF-α, tumor necrosis factor α; IL, interleukin; LPS, lipopolysaccharide; T-AOC, total antioxidant capacity; MDA, malondialdehyde; SOD, superoxide dismutase; GSH-Px, glutathione peroxidase.

Table S13 Effects of gut microbiota transplantation from donor mice fed CON, Cd, and Mel +Cd on the villus morphology, barrier function, anti-oxidative capacity, LPS and cytokine concentrations in the ileum, and DAO activity and D-lactic acid concentration in the serum in antibiotics-treated mice for 10 weeks [a].

Item[b]	GMT (CON)	GMT (Cd)	GMT (Mel+Cd)	SEM	P value
**Ileal villus morphology**					
Villous height, μm	263.98[a]	163.56[c]	205.84[b]	6.49	0.016
Crypt depth, μm	100.12[c]	143.42[a]	119.99[b]	5.34	0.009
VCR	2.64[a]	1.14[c]	1.72[b]	0.15	0.014
**Ileal barrier function**					
TER, $\Omega \cdot cm^2$	53.37[a]	32.47[c]	41.65[b]	4.68	0.007
FD4 flux, $\mu g \cdot cm^{-2} \cdot h^{-1}$	1.78[c]	3.04[a]	2.24[b]	0.12	0.015
**Ileal anti-oxidative capacity**					
GSH-Px, U/mg prot	30.26[a]	12.59[c]	19.47[b]	1.28	0.006
SOD, U/mg prot	41.12[a]	19.95[c]	30.17[b]	1.65	0.009
T-AOC, U/mg prot	5.24[a]	3.15[c]	4.28[b]	0.17	0.008
MDA, nmol/mg prot	1.02[c]	1.75[a]	1.34[b]	0.09	0.021
**Ileal LPS and cytokine concentrations**					
LPS, ng/mg prot	2.47[c]	5.11[a]	3.76[b]	0.11	0.009
TNF-α, ng/mg prot	6.25[c]	10.17[a]	8.09[b]	0.58	0.018
IL-1β, ng/mg prot	7.89[c]	13.94[a]	10.11[b]	0.71	0.015
IL-6, ng/mg prot	9.47[c]	17.89[a]	12.79[b]	0.48	0.008
**Serum**					
DAO, $U\ mL^{-1}$	1.07[c]	1.67[a]	1.40[b]	0.09	0.007

D-lactic acid, mg L^{-1}	0.67^{c}	1.31^{a}	1.01^{b}	0.06	0.011

[a] Mean value with their standard errors of the mean (SEM) (n = 12 per group). Within a row, mean values without a common letter differ (*P* < 0.05).

[b] GMT, gut microbiota transplantation; GMT(CON), the microbiota-depleted mice that were originally fed control diet received microbiota transplantations from donor mice fed control diet; GMT(Cd), the microbiota-depleted mice that were originally fed control diet received microbiota transplantations from donor mice treated $CdCl_2$; GMT (Mel+Cd), the microbiota-depleted mice that were originally fed control diet received microbiota transplantations from donor mice in Mel + Cd groups; VCR, villous height/crypt depth ratio; DAO, diamine oxidase; TER, transepithelial electrical resistance; FD4, fluorescein isothiocyanate dextran (4 kDa); TNF-α, tumor necrosis factor α; IL, interleukin; LPS, lipopolysaccharide; T-AOC, total antioxidant capacity; MDA, malondialdehyde; SOD, superoxide dismutase; GSH-Px, glutathione peroxidase.

Table S14 Effects of melatonin and $CdCl_2$ oral gavage on the villus morphology, barrier function, anti-oxidative capacity, LPS and cytokine concentrations in the ileum, and DAO activity and D-lactic acid concentration in the serum in antibiotics-treated mice for 10 weeks [a].

Item[b]	Anti (CON)	Anti (Cd)	Anti (Mel+Cd)	SEM	P value
**Ileal villus morphology**					
Villous height, μm	252.88	247.36	251.76	7.43	0.116
Crypt depth, μm	94.74	99.16	92.26	3.74	0.207
VCR	2.67	2.50	2.73	0.31	0.109
**Ileal barrier function**					
TER, $\Omega\cdot cm^2$	51.43	49.94	50.69	4.11	0.104
FD4 flux, $\mu g\cdot cm^{-2}\cdot h^{-1}$	1.89	1.91	1.86	0.13	0.192
**Ileal anti-oxidative capacity**					
GSH-Px, U/mg prot	26.45	25.69	26.08	1.85	0.118
SOD, U/mg prot	38.11	37.96	38.03	1.47	0.241
T-AOC, U/mg prot	5.01	4.95	4.99	0.13	0.112
MDA, nmol/mg prot	1.25	1.19	1.22	0.05	0.097
**Ileal LPS and cytokine concentrations**					
LPS, ng/mg prot	2.21	2.19	2.17	0.05	0.105
TNF-α, ng/mg prot	5.18	5.23	5.19	0.36	0.089
IL-1β, ng/mg prot	7.14	7.19	7.12	0.47	0.129
IL-6, ng/mg prot	9.13	9.18	9.14	0.68	0.117
**Serum**					
DAO, U mL^{-1}	0.95	1.01	0.99	0.07	0.223

D-lactic acid, mg L^{-1}	0.77	0.80	0.81	0.09	0.096

[a] Mean value with their standard errors of the mean (SEM) (n = 12 per group). Within a row, mean values without a common letter differ (*P* < 0.05).

[b] Anti, antibiotics; Anti (CON), the microbiota-depleted mice that were originally fed control diet; Anti (Cd), the microbiota-depleted mice that were originally fed control diet treated $CdCl_2$; Anti (Mel+Cd), the microbiota-depleted mice that were originally fed control diet treated melatonin and $CdCl_2$; VCR, villous height/crypt depth ratio; DAO, diamine oxidase; TER, transepithelial electrical resistance; FD4, fluorescein isothiocyanate dextran (4 kDa); TNF-α, tumor necrosis factor α; IL, interleukin; LPS, lipopolysaccharide; T-AOC, total antioxidant capacity; MDA, malondialdehyde; SOD, superoxide dismutase; GSH-Px, glutathione peroxidase.

Table S15 Effects of melatonin supplementation on SCFAs and LPS concentrations in colonic contents in $CdCl_2$ treated mice for 10 weeks[a].

Item[b]	CON	Cd	Mel+Cd	SEM	P value
LPS, ng/mL	39.27^c	72.48^a	54.67^b	2.86	0.009
Acetate, μg/g	139.11	142.17	136.34	6.16	0.108
Propionate, μg/g	80.91^a	49.16^c	62.91^b	2.11	0.006
Butyrate, μg/g	52.31^a	32.18^c	43.09^b	1.28	0.011
Isobutyrate, μg/g	11.37	11.98	11.07	0.89	0.207
Valerate, μg/g	9.68^a	5.32^c	7.02^b	0.54	0.006
Isovalerate, μg/g	8.47	8.04	8.36	0.47	0.231

[a] Mean value with their standard errors of the mean (SEM) (n = 12 per group). Within a row, mean values without a common letter differ ($P < 0.05$).

[b] CON, mice in the control group fed a standard lab diet; Cd group, mice were challenged with $CdCl_2$ once a day by gavage; Mel+Cd group, the $CdCl_2$ mice were treated with melatonin; SCFAs, short-chain fatty acids; LPS, lipopolysaccharide.

Table S16 Effects of gut microbiota transplantation from donor mice fed CON, Cd, and Mel+Cd on SCFAs and LPS concentrations in colonic contents in antibiotics-treated mice for 10 weeks [a].

Item[b]	GMT (CON)	GMT (Cd)	GMT (Mel+Cd)	SEM	P value
LPS, ng/mL	30.11[c]	59.68[a]	43.36[b]	2.13	0.006
Acetate, µg/g	123.57	127.63	122.31	5.08	0.204
Propionate, µg/g	69.46[a]	38.36[c]	50.15[b]	1.99	0.013
Butyrate, µg/g	46.31[a]	32.56[b]	38.15[ab]	1.48	0.024
Isobutyrate, µg/g	9.85	9.67	9.71	0.59	0.183
Valerate, µg/g	7.96[a]	5.58[c]	6.62[b]	0.49	0.015
Isovalerate, µg/g	8.02	7.96	8.04	0.39	0.193

[a] Mean value with their standard errors of the mean (SEM) (n = 12 per group). Within a row, mean values without a common letter differ ($P < 0.05$).

[b] GMT, gut microbiota transplantation; GMT(CON), the microbiota-depleted mice that were originally fed control diet received microbiota transplantations from donor mice fed control diet; GMT(Cd), the microbiota-depleted mice that were originally fed control diet received microbiota transplantations from donor mice treated $CdCl_2$; GMT (Mel+Cd), the microbiota-depleted mice that were originally fed control diet received microbiota transplantations from donor mice in Mel + Cd groups; LPS, lipopolysaccharide; SCFAs, short-chain fatty acids.

Table S17 Effects of melatonin and $CdCl_2$ oral gavage on SCFAs and LPS concentrations in colonic contents in antibiotics-treated mice for 10 weeks [a].

Item[b]	Anti (CON)	Anti (Cd)	Anti (Mel+Cd)	SEM	P value
LPS, ng/mL	26.15	28.43	25.72	2.17	0.203
Acetate, µg/g	119.57	112.69	116.53	7.83	0.105
Propionate, µg/g	73.23	70.35	74.29	2.78	0.078
Butyrate, µg/g	39.13	36.63	37.48	1.95	0.093
Isobutyrate, µg/g	8.95	9.02	8.92	0.59	0.116
Valerate, µg/g	7.06	7.02	6.95	0.39	0.089
Isovalerate, µg/g	6.96	7.01	6.94	0.51	0.115

[a] Mean value with their standard errors of the mean (SEM) (n = 12 per group). Within a row, mean values without a common letter differ ($P < 0.05$).

[b] Anti, antibiotics; Anti (CON), the microbiota-depleted mice that were originally fed control diet; Anti (Cd), the microbiota-depleted mice that were originally fed control diet treated $CdCl_2$; Anti (Mel+Cd), the microbiota-depleted mice that were originally fed control diet treated melatonin and $CdCl_2$; LPS, lipopolysaccharide; SCFAs, short-chain fatty acids.
